# Induction of Noxa-Mediated Apoptosis by Modified Vaccinia Virus Ankara Depends on Viral Recognition by Cytosolic Helicases, Leading to IRF-3/IFN-β-Dependent Induction of Pro-Apoptotic Noxa

**DOI:** 10.1371/journal.ppat.1002083

**Published:** 2011-06-16

**Authors:** Pedro Eitz Ferrer, Stephanie Potthoff, Susanne Kirschnek, Georg Gasteiger, Wolfgang Kastenmüller, Holger Ludwig, Stefan A. Paschen, Andreas Villunger, Gerd Sutter, Ingo Drexler, Georg Häcker

**Affiliations:** 1 Institute of Medical Microbiology, Immunology and Hygiene, Technische Universität München, Munich, Germany; 2 Institute of Medical Microbiology and Hygiene, University Freiburg, Freiburg, Germany; 3 University of Freiburg, Faculty of Biology, Freiburg, Germany; 4 Institute of Virology and Clinical Cooperation Group “Antigen-specific Immunotherapy”, TechnischeUniversitätMünchen and Helmholtz ZentrumMünchen, Munich, Germany; 5 Division of Virology, Paul-Ehrlich-Institut, Langen, Germany; 6 Division of Developmental Immunology, Biocenter, Innsbruck Medical University, Innsbruck, Austria; 7 Institute for Infectious Diseases and Zoonoses, Ludwig-Maximilians-Universität, Munich, Germany; University of Florida, United States of America

## Abstract

Viral infection is a stimulus for apoptosis, and in order to sustain viral replication many viruses are known to carry genes encoding apoptosis inhibitors. F1L, encoded by the orthopoxvirus modified vaccinia virus Ankara (MVA) has a Bcl-2-like structure. An MVA mutant lacking F1L (MVAΔF1L) induces apoptosis, indicating that MVA infection activates and F1L functions to inhibit the apoptotic pathway. In this study we investigated the events leading to apoptosis upon infection by MVAΔF1L. Apoptosis largely proceeded through the pro-apoptotic Bcl-2 family protein Bak with some contribution from Bax. Of the family of pro-apoptotic BH3-only proteins, only the loss of Noxa provided substantial protection, while the loss of Bim had a minor effect. In mice, MVA preferentially infected macrophages and DCs *in vivo*. In both cell types wt MVA induced apoptosis albeit more weakly than MVAΔF1L. The loss of Noxa had a significant protective effect in macrophages, DC and primary lymphocytes, and the combined loss of Bim and Noxa provided strong protection. Noxa protein was induced during infection, and the induction of Noxa protein and apoptosis induction required transcription factor IRF3 and type I interferon signalling. We further observed that helicases RIG-I and MDA5 and their signalling adapter MAVS contribute to Noxa induction and apoptosis in response to MVA infection. RNA isolated from MVA-infected cells induced Noxa expression and apoptosis when transfected in the absence of viral infection. We thus here describe a pathway leading from the detection of viral RNA during MVA infection by the cytosolic helicase-pathway, to the up-regulation of Noxa and apoptosis via IRF3 and type I IFN signalling.

## Author Summary

Viruses have come up with a diverse set of mechanisms to stop infected cells from committing suicide and hence secure their own propagation. In this study we use the DNA virus Modified Vaccinia virus Ankara, a highly attenuated version Vaccinia Virus, to study how cells detect viral infection and induce apoptosis. Modified Vaccinia virus Ankara is currently in clinical trials for its use in various vaccination protocols. By using a broad array of immortalized and primary cell types we observed that viral infection induced programmed cell death was controlled by proteins predominantly involved in detection of viral RNA, in particular proteins involved in the type 1 interferon response. The novelty of our findings lies on the observation that not only can RNA from DNA viruses be detected and activate the type 1 interferon response to infection, but that these responses can also directly modulate the levels of proteins regulating programmed cell death. Future treatments of infections by viral pathogens could exploit the synergistic ability of the type 1 interferon responses and programmed cell death in order to inhibit viral propagation.

## Introduction

Cell death by apoptosis can protect a multicellular organism against viral infection: if the first infected cell dies in time the virus will not have the chance of producing new viral particles. Many viruses counter this cellular response by carrying genes coding for inhibitors of the host cell's apoptosis machinery. This interrelationship has first been shown clearly in a baculovirus where the loss of the caspase-inhibitor p35 leads to cell death and reduction in viral productivity [1]. Numerous viral anti-apoptotic genes have by now been found, whose products often have structural resemblance to mammalian anti-apoptotic proteins [2], [3].

A number of poxviruses carry, among other anti-apoptotic genes, genes coding for proteins that resemble the inhibitory members of the mammalian Bcl-2-family of proteins. These genes have very little (if any) primary sequence homology with the mammalian proteins but crystallization studies have shown that they adopt a virtually identical structure. One critical feature of anti-apoptotic function of Bcl-2-proteins is the existence of a hydrophobic groove that accommodates the BH3-domain of pro-apoptotic Bcl-2-family members and thereby inhibits apoptosis [4].

Intriguingly, there is considerable variation at this critical structural position in poxviral Bcl-2-related proteins. The M11L protein from myxoma virus can bind BH3-domains with high affinity and cell biological studies indicate that the binding to pro-apoptotic Bax and Bak (via their BH3-domains) is necessary for the anti-apoptotic function of M11L [5]. Other poxviral proteins with a Bcl-2-like fold (as determined in the crystal structure) lack the structural hydrophobic groove and hence the ability to bind BH3-domains, despite having the other structural features of Bcl-2 [6]. These proteins (and other, related poxvirus-encoded proteins) seem to have functions in manipulating the host immune response rather than in apoptosis prevention [7]. How these structures and diverging functions have evolved is intriguing but unclear.

The F1L protein has been found to inhibit apoptosis in vaccinia virus (VACV) as well as in the highly attenuated VACV strain modified vaccinia virus Ankara (MVA) [8], [9]. The crystal structure of MVA F1L surprisingly showed that F1L protein dimerizes through a helical domain swap and that it, although it can bind some BH3-domain-peptides, does so with low affinity. Even the binding to the BH3-domain of Bim, the pairing of F1L with the highest affinity among all BH3-domains, occurs at a much lower affinity than the binding of any BH3-domain to mammalian Bcl-2 proteins thought to be functionally relevant [10], [11]. F1L protein has further been identified as an inhibitor of caspase-9 [12].

Mammalian anti-apoptotic Bcl-2-like proteins (Bcl-2, Bcl-X_L_, Bcl-W, Mcl-1, A1) can inhibit most forms of apoptosis, namely those using the release of cytochrome *c* from mitochondria as a signalling step. Cytochrome *c* is normally localised in the mitochondrial intermembrane space. Once released to the cytosol it initiates the formation of a big protein complex known as the apoptosome; in the apoptosome caspases are activated [13].

The release of cytochrome *c* is achieved by the action of the two pro-apoptotic effectors of the Bcl-2-family, Bax and Bak, either one alone or both together. Bax/Bak in turn are activated by one or several of the Bcl-2-family subclass of BH3-only proteins (Bim, Bid, Puma, Noxa, Bmf, Bad, Bik, Hrk). The anti-apoptotic Bcl-2-like proteins can bind (although with varying affinity) both BH3-only proteins and Bax/Bak and probably inhibit apoptosis by this direct binding; whether they inhibit apoptosis preferentially by binding to BH3-only proteins or to Bax/Bak is under dispute. The other BH3-only proteins very likely others operate indirectly by inhibiting the suppressors (Bcl-2 and Bcl-2-like inhibitors) [4], [14].

Given its anti-apoptotic function and Bcl-2-like structural fold it is very likely that F1L also inhibits apoptosis by binding of pro-apoptotic Bcl-2 family proteins. (VACV) F1L binding to Bak (but not Bax) has been demonstrated in immuno-precipitation experiments from infected cells [15], [16] and this interaction has been mapped within the protein [17]. It has also been suggested that F1L directly binds Bim [18]. However, even the combined inhibition of Bim and Bak is unlikely to explain the profound inhibition of apoptosis in VACV- or MVA-infected cells [8], [9].

F1L-deficient VACV and MVA induce Bax/Bak-dependent apoptosis in their host cells [9], [17] suggesting that viral infection activates BH3-only proteins, which then activate Bax/Bak. Bim appears to play some role as Bim-deficient cells undergo slightly less apoptosis than wt cells when infected with VACV-ΔF1L [18]. Bad has also been proposed to play a role in VACV detection as MEK induced activation of Bad has been shown to be inhibited by VACV secreted VGF protein [19]. The receptors that an infected cell uses to detect the viral infection and that then activate the apoptotic apparatus, and which components of the apoptotic machinery (besides Bim) are involved in transmitting this signal are unknown.

In this study we analysed these upstream signals. We found that both Bim and (more importantly) Noxa were involved in the induction of apoptosis by MVAΔF1L. Analysis of the upstream signals suggested a contribution from the type 1 interferon (IFN) signalling pathway in MVA-induced activation of the apoptotic pathway.

## Results

**Figure 1:**
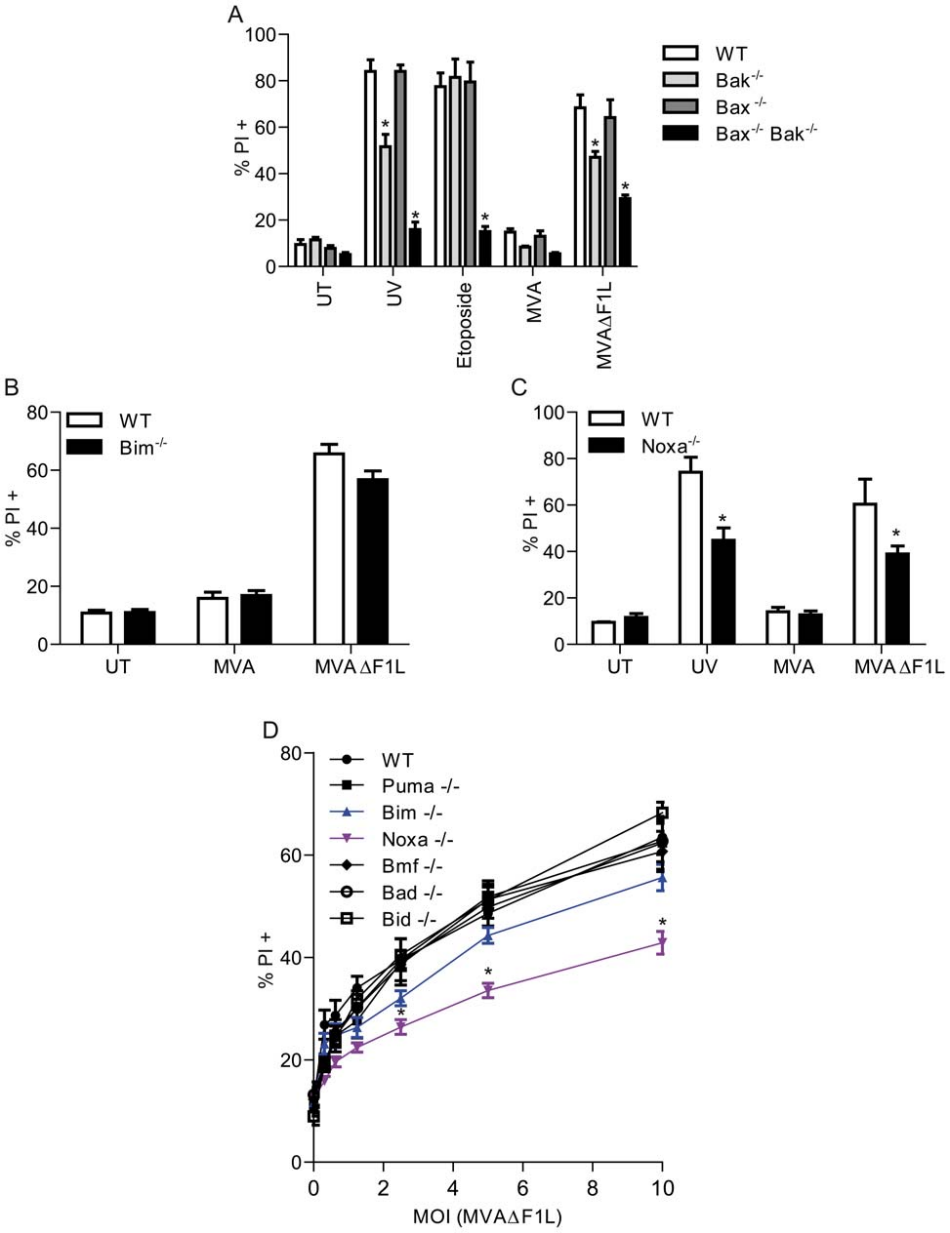
MVAΔF1L mediated apoptosis is predominantly induced by the BH3-only proteinNoxa. (A) wt, Bak^−/−^, Bax^−/−^, and Bax^−/−^/Bak^−/−^ MEFs were infected with MVA or MVAΔF1L (M.O.I = 10) for 20 h. Cell death was assessed by PI staining. UT, untreated (B) wt and Bim^−/−^ MEFs were infected as in A. (C) wt and Noxa^−/−^ MEFs were infected as in A. In parallel, aliquots from the same cell lines were treated with UV light (100 J/m^2^) and incubated for 20 h. (D) wt, Bim^−/−^, Noxa^−/−^, Bmf^−/−^, Bad^−/−^, Bid^−/−^, and Puma^−/−^ MEFs were treated as in A. (* indicates statistical significance (Student's t-test, p≤0.05 with data indicating mean/SEM of n≥3).

For these studies we used infection of various cell types and cells from genetically modified mice for analysis with either wt MVA or with the deletion mutant virus MVAΔF1L, which lacks the F1L gene. Expression of the F1L protein in infected cells was detected from 8 through to 48 h (Supplementary Figure S1). Upon infection of mouse embryonic fibroblasts (MEFs), cell death was first detectable around 8–12 h post-infection (Supplementary Figure S2A). As reported earlier [9], infection with MVAΔF1L induced apoptosis via the mitochondrial pathway. Mitochondrial apoptosis depends on the activities of either Bax or Bak. MVAΔF1L induced apoptosis was reduced in Bak-deficient MEFs and abolished in Bax/Bak-double-deficient MEFs (Figure 1A, Supplementary Figure S2B) much like it has previously been reported for UV mediated apoptotic stress [20]. These results suggest that infection with MVA mainly triggers Bak but in its absence also Bax, whereas F1L blocks this process (similar results have been reported previously for MVA by us [9] and for VACV by others [15], [16]).

Bax/Bak are activated by the action of BH3-only proteins. To test the contribution of individual BH3-only proteins we tested MEFs from mice deficient in individual BH3-only proteins for differences in apoptosis when infected with MVAΔF1L. As shown in Figure1B and Supplementary Figure S2C, the loss of Bim conferred a small degree of protection; a similar effect has previously been reported for VACVΔF1L [18], [19]. No protection was seen by individual loss of Puma, Bid, Bad or Bmf (Figure 1D, Supplementary Figure S2D) unlike previously reported for VACVΔF1L, where Bad-specific RNAi strongly inhibited apoptosis induced by infection of HeLa cells [19]. However, MEFs deficient in Noxa were clearly protected against apoptosis induced by infection with MVAΔF1L, and this effect was stronger than the one seen in Bim-deficient cells (Figure 1C, D, Supplementary Figure S2C).

In earlier work we had found that there was no effect of the loss of individual BH3-only proteins during infection with MVAΔF1L (results mentioned as data not shown in [9]). We are unable to retrace what it was that produced this incorrect result at the time.

**Figure 2:**
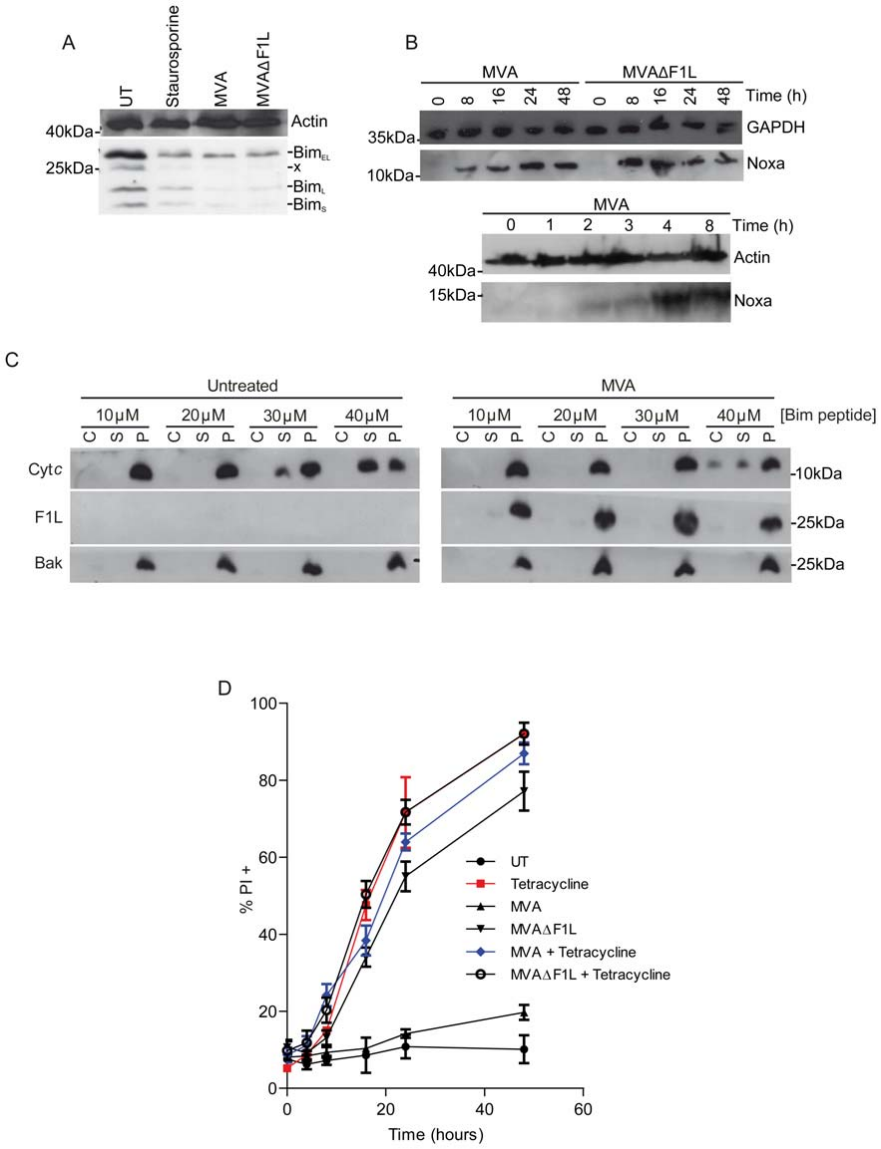
MVA infection causes a reduction in Bim levels and induction of Noxa expression while F1L weakly inhibits Bim induced apoptosis. (A) HeLa cells were treated with staurosporine (1 µM) for 8 h or infected with MVA or MVAΔF1L (M.O.I = 10) for 20 h and Bim levels were assessed by Western blotting (x represents protein of unknown origin). (B) HeLa cells were infected with MVA or MVAΔF1L (M.O.I = 10) for the indicated times and Noxa levels were assessed by Western blotting. (C) Digitonin (0.025% w/v) permeabilized cells from uninfected or MVA infected MEFs (8 h p.i) were treated with the indicated concentrations of Bim BH3 peptide to detect cytochrome *c* release by Western blot. C corresponds to the cytosolic fraction released upon cellular membrane permeabilization. S corresponds to the supernantant fraction after Bim-BH3-peptide treatment of permeabilised cells. P represents the pellet fraction including nucleus, cellular membrane and intact or treatment-permeabilized mitochondria. (D) MEFs carrying tetracyclin inducible Bim_s_ were infected with MVA or MVAΔF1L for 8 h before addition of tetracycline (0.1 µg/ml) for 12 h. Apoptosis was assessed by PI staining (* indicates statistical significance according to the student's t-test, p≤0.05 with data indicating mean/SEM of n≥3; x indicates unspecific band).

Noxa protein is typically expressed only at low levels but is induced by pro-apoptotic stimuli. We were unable to detect Noxa protein in MEFs (which is most likely due to the low sensitivity of antibodies against mouse Noxa by Western blot) and therefore used the human epitheloid cell line HeLa to test for Bim and Noxa-expression during MVA-infection. Bim protein levels decreased within 20 h.p.i, perhaps explaining the observation that Bim only plays a minor role during MVA or MVAΔF1L infection (Figure 2A). Loss of Bim protein was also observed in MEFs upon infection (data not shown). Infection with either MVA or MVAΔF1L led to the induction of Noxa protein in HeLa cells, beginning at about 4 h p. i. (Figure 2B). Expression of F1L occurred with a similar time course (Supplementary Figure S1). This suggests that the infection by MVA induces the expression of Noxa, which is required for full induction of apoptosis, while F1L inhibits Noxa-dependent apoptosis. Noxa and, to a lesser extent, Bim are therefore triggers of apoptosis induced by MVAΔF1L.

Although these data indicate that F1L blocks Bim- and Noxa-dependent apoptosis, it is not clear how this works on a molecular level. It has been suggested that F1L directly binds to and inhibits Bim [10], [18]. We tested this hypothesis in two separate experimental systems. In the first of those, membrane fractions including intact mitochondria from uninfected control cells and from cells infected with MVA (the latter containing F1L protein) were incubated with a synthetic peptide comprising the Bim BH3-domain (the active domain in BH3-only proteins). This leads to the activation of Bax and Bak and the release of cytochrome *c* [21], [22], [23]. In mitochondria isolated from control cells, Bim-peptide caused substantial release of cytochrome *c* at concentrations starting around 30 µM (similar to results from other studies, Figure 2C). The release of cytochrome *c* from mitochondria from MVA-infected cells, and hence expressing F1L, was achieved at slightly higher concentrations of Bim peptide (less than a 2-fold) (Figure 2C).

In the second experimental setting, we used a MEF cell line where Bim_S_ (the most active splice form of Bim) is placed under the control of a tetracycline-inducible promoter. Addition of tetracycline to the culture medium induces Bim in these cells and causes rapid apoptosis (Supplementary Figure S3A, B). Prior infection with MVA for 8 h, to ensure F1L expression, again had only a very small protective effect in this system (Figure 2D, Supplementary Figure S3A, C). While overexpression of Bim_s_ is not reduced during MVA infection, endogenous Bim (Bim_EL_) levels appear to be lower in MVA infected cells, as observed in Helas (Supplementary Figure S3B, Figure 2A). These results suggest that direct binding of F1L to Bim, which can occur with low affinity [10], plays only a minor role in the protection against virus induced apoptosis by F1L in these cells.

**Figure 3:**
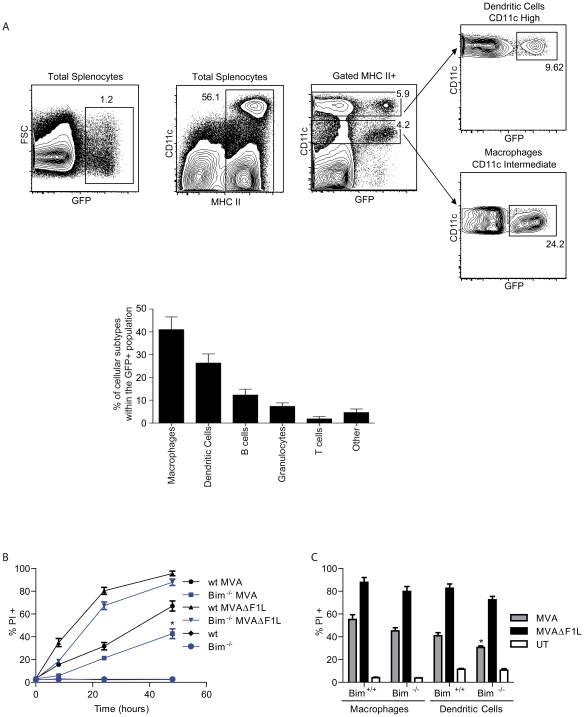
Infection pattern and effect of the loss of Bim in primary haematopoietic cells upon infection of mice with MVA. (A) Mice (n  =  3) were infected i.v. with 3×10^8^ IU of MVA-GFP for 3 hours before spleens were harvested. Splenocytes were stained and cell populations were identified as follows: macrophages (MHC II high /CD11c intermediate / CD11b high), dendritic cells (MHC II high /CD11c high), B cells (MHC II high-intermediate /CD45R (B220) high), granuloytes (Gr-1 high / CD11b high), T cells (CD3+). Dot blots show the gating strategy for macrophages and dendritic cells from total splenocytes. Histogram gives the relative distribution of the different cell types within the total number of MVA-infected (GFP+) cells. (B) wt and Bim^−/−^ M-CSF bone marrow derived macrophages were infected with MVA or MVAΔF1L at an M.O.I of 10 for the indicated times. Cell death was assessed by PI staining. (C) wt and Bim^−/−^ M-CSF bone marrow derived macrophages or GM-CSF bone marrow derived dendritic cells where infected as in B for 20 h p.i. UT, untreated (* indicates statistical significance according to the student's t-test, p≤0.05 with data showing mean/SEM of n≥3).

Cell lines and MEFs are versatile tools for the analysis of MVA-infection *in vitro* but are not the natural host cells of MVA-infection *in vivo*. Infection of mice with a recombinant MVA driving the expression of GFP in infected cells showed that upon intravenous infection especially macrophages and dendritic cells (DCs) and to a lesser extent lymphocytes were infected in the spleen (Figure 3A; Supplementary Figure S4 shows results at a 10-fold higher dosage of virus). We therefore undertook an analysis of the apoptotic response of these host cells to infection with MVA or MVAΔF1L.

Mouse myeloid macrophages and DCs were differentiated from bone marrow. DCs differ from epithelial cells and fibroblasts in that they undergo apoptosis upon infection with wt MVA [24], [25]. We found substantial induction of apoptosis in both macrophages and DCs upon infection with MVA (Figure 3B, C). However, apoptosis induced by MVAΔF1L was much faster and reached higher levels over 48 h (Figure 3B, C). As we had found for fibroblasts, the loss of Bim gave small protection against apoptosis induced by MVA or by MVAΔF1L in both cell types (Figure 3B, C).

**Figure 4:**
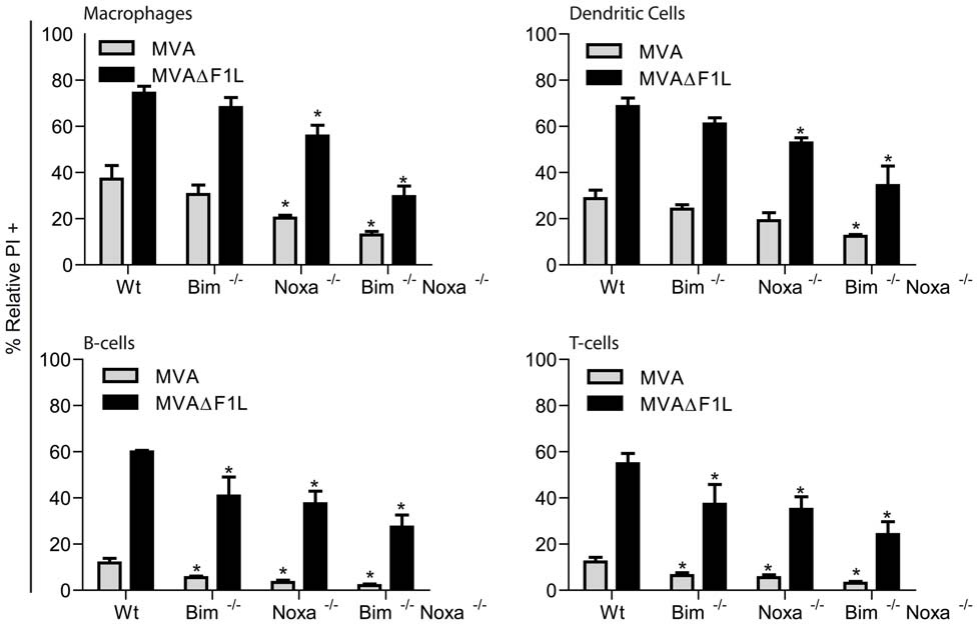
Contributions of Noxa and Bim to apoptosis induced by MVA and MVAΔF1L in primary MVA target cells. M-CSF bone marrow derived macrophages and GM-CSF bone marrow derived dendritic cells, B220^+^ MACS sorted B-cells and NK1.1^−^/B220^−^/MHCII^−^ MACS sorted ConA activated T-cells were infected with MVA or MVAΔF1L at an M.O.I of 10 for 20 h. Cell death was assessed by PI staining and relative numbers were calculated by calculating the % increase in PI proportional to the untreated sample with the following equation: (((% treated PI^+^)−(% untreated PI^+^))/% untreated PI^+^*100). (* indicates statistical significance according to the student's t-test, p≤0.05; with data giving mean/SEM of n≥3).

We then tested apoptosis induction in macrophages, DCs, activated T-cells and B cells isolated from wt, Bim-, Noxa- or Bim/Noxa-deficient mice. MVAΔF1L induced substantial apoptosis in all these cell types while MVA-induced apoptosis reached higher levels only in the two myeloid cell types (DC, macrophages) but not in lymphocytes (Figure 4, Supplementary Figure S5 and S6). There were small differences in the pattern of apoptosis-sensitivity in the various cell types. The protection against MVAΔF1L induced apoptosis by the loss of Bim was minimal in myeloid but more clearly detectable in lymphoid cells. Single loss of Noxa provided moderate protection in all cell types, and the combined loss of Bim and Noxa protected not completely but very strongly against apoptosis induced by MVAΔF1L infection in all cells analysed (Figure 4, S5, S6).

To ensure that the decrease in apoptosis observed due to loss of Bim and to a greater extent loss of Noxa was not specific for mouse, we tested the role of BH3-only proteins in HeLa cells by RNAi. siRNAs directed against Bim, Puma or Noxa were transfected into HeLa cells prior to infection, alone or in combination. As shown in Figure S6, there was no detectable protection by knock-down of Bim, Puma or the combination of Bim and Puma. However, Noxa-specific RNAi strongly reduced apoptosis induced by MVAΔF1L infection and inhibited the strong induction of Noxa induced by MVAΔF1L infection in these cells (Supplementary Figure S7B, lanes 3, 5 and 8). The combination of Bim- and Noxa-knock-down failed to give more protection than RNAi against Noxa alone, suggesting that Bim played only a very minor role in these cells, as previously reported for VACVΔF1L [19].

These data suggested that the induction of Noxa protein upon infection with MVAΔF1L was a major triggering event of apoptosis. Noxa was originally identified as a p53 transcriptional target. However, macrophages lacking expression of p53 did not show a significant decrease in apoptosis in response to MVA or MVAΔF1L (Supplementary Figure S8A). Subsequent work has shown that the Noxa promoter is also regulated by a CreB binding site and an interferon-stimulated responsive element (ISRE) [26], and that Noxa is induced by IFN I. INFβ was clearly induced during MVA and MVAΔF1L infection (Supplementary Figure S8B), as was IL-18, a cytokine whose secretion is regulated through the inflammasome (Supplementary Figure S8C). However, loss of the inflammasome activity by ASC deficiency had no effect on the apoptotic response of macrophages to MVA or MVAΔF1L (Supplementary Figure S8D). Since IFN I are induced during MVA and MVAΔF1L infection within 8 h we focussed on the investigation of this system and its role in Noxa-induction and apoptosis during infection with MVAΔF1L.

**Figure 5:**
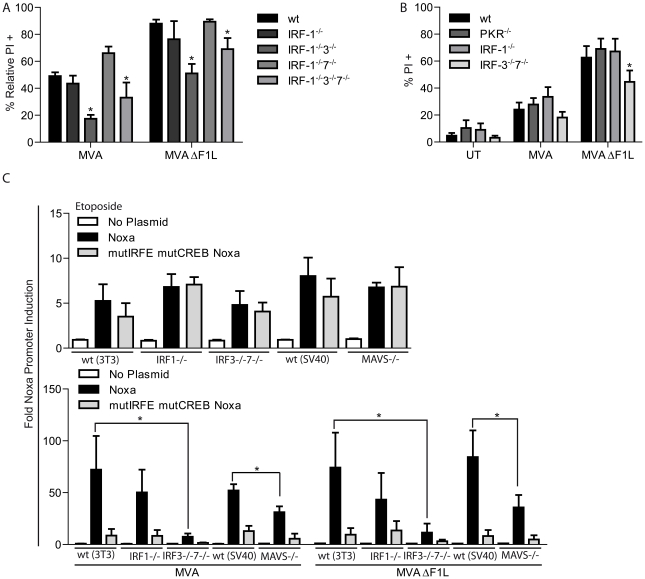
A, B, MVA induced apoptosis requires IRF-3 but not IRF-1, -7 or PKR. (A) wt, IRF-1^−/−^, IRF-1^−/−^3^−/−^, IRF-1^−/−^7^−/−^ and IRF-1^−/−^3^−/−^7^−/−^ M-CSF bone marrow derived macrophages were infected with MVA or MVAΔF1L at an M.O.I of 10 for 20 h. Cell death was assessed by PI staining and relative numbers were calculated by calculating the % increase in PI proportional to the untreated sample as in Figure 4. (B) wt, PKR^−/−^, IRF-1^−/−^ and IRF-3^−/−^7^−/−^ MEFs were infected as in A. Cell death was assessed by PI staining. UT, untreated. (C) **Infection with MVA or MVAΔF1L induces Noxa-promoter activity through IRF-3.** wt, IRF-1^−/−^, IRF-3^−/−^7^−/−^ and MAVS^−/−^ MEFs were transfected with the reporter constructs of the *NOXA* promoter (pGL3-basic plasmid carrying wt or a double mutant (deletion of the CreB and IRE sites) Noxa 1.2 kb promoter region). 24 h after transfection cells were split onto a 96 well plate. The next day aliquot wells were treated with etoposide (10 µM) or infected with MVA or MVAΔF1L (M.O.I  =  10). 8 h later, luciferase activity was measured and the index of induction was calculated from the ratio of the luciferase activity in treated cells divided by the luciferase activity in untreated cells. (* indicates significance according to the student's t-test, p≤0.05 with data giving mean/SEM of n≥3).

We first isolated and tested macrophages from mice deficient in various interferon responsive factors (IRFs; transcription factors involved in the regulation of IFN and IFN-responsive genes). There was significant protection against apoptosis induced by either MVA or MVAΔF1L by the combined loss of IRF-1 and -3 or IRF-1, -3 and -7 but not the loss of IRF-1 or IRF-1 and -7 (Figure 5A, S6B; mice deficient only in IRF-3 were not available to us). IRF-3 is therefore required for the full induction of apoptosis by infection with MVA/MVAΔF1L.

IRF-3 is activated by signalling pathways that can be triggered by the recognition of viral molecules. In particular, viral nucleic acids can be recognized by a number of receptors including protein kinase R (PKR; recognizing double stranded RNA), cytosolic helicases (RIG-I, MDA5; recognizing free 5′-triphosphates and double stranded RNA, respectively) and Toll-like receptors (TLR).

We first tested MEFs from mice deficient in protein kinase R (PKR) but found no protection while MEFs double deficient in IRF-3 and -7 were significantly protected (Figure 5B). We also tested macrophages deficient in MyD88 (a major adaptor molecule in the Toll-like receptor signalling pathway) and observed that there was no protection in response to MVA or MVAΔF1L induced apoptosis (Supplementary Figure S8D).

We then turned to the analysis of the signalling pathways that induce Noxa-promoter activity. A luciferase reporter construct containing either 1.2 kb Noxa promoter-sequence (carrying an ISRE and bindings sites for p53 and creB), or the same sequence lacking the binding sites for creB and IRFs [26] were transfected into MEFs from mice with various gene deletions. These cells were then either treated with etoposide (known to induce Noxa via p53) or infected with MVA or MVAΔF1L.

Etoposide induced Noxa-promoter activity in cells from all genotypes tested and this induction was similar in the cases of the intact and mutant promoters (which still contains the p53 recognition site), consistent with the p53-dependent induction of Noxa by etoposide (Figure 5C). The induction of Noxa-promoter activity by infection with MVA or MVAΔF1L, however, was almost abrogated by the promoter deletions (Figure 5C). This indicates that the Noxa induction is almost completely achieved via the IFN I system.

The activity of the intact promoter upon infection was normal in IRF-1-deficient cells but strongly diminished in cells deficient in IRF-3 and -7, again consistent with an important role of IRF-3 in virus-induced up-regulation of Noxa. When we tested cells deficient in the mitochondrial helicase adapter MAVS (also known as IPS-1, VISA or CARDIF) we also found a significant reduction in Noxa-promoter induction upon infection with MVA or MVAΔF1L (Figure 5C).

**Figure 6:**
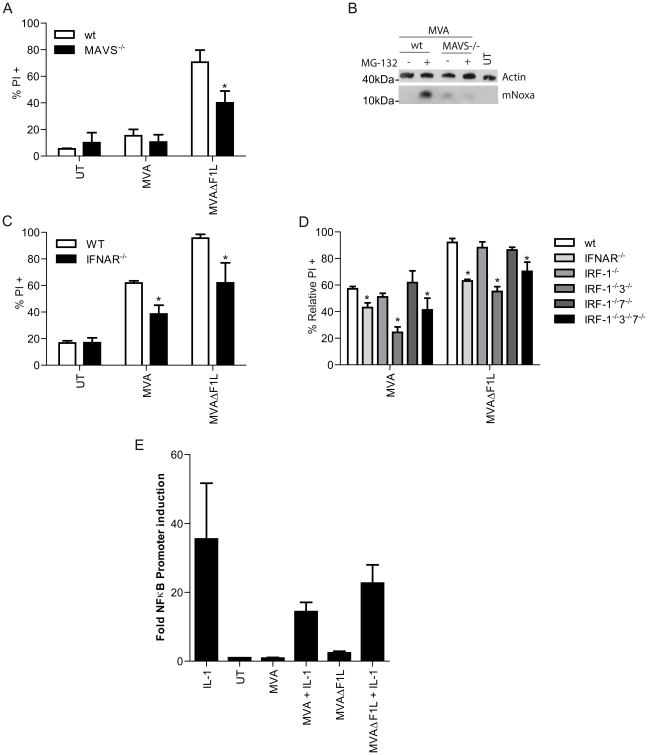
IRF3 induced activation of Noxa is dependent on MAVS and IFN-I-signaling. (A) wt and MAVS^−/−^ MEFs were infected with MVA or MVAΔF1L at an M.O.I of 10 for 20 h. Cell death was assessed by PI staining. (B) Cells were infected as in A but 16 hp.i cells remained untreated or were treated with MG-132 (40 µM) for a further 4 h. Mouse Noxa (mNoxa) was detected by Western blot. (C) wt and IFNAR^−/−^ M-CSF bone marrow derived macrophages were infected with MVA or MVAΔF1L at an M.O.I of 10 for 20 h. Cell death was assessed by PI staining. (D) wt, IFNAR^−/−^, IRF-1^−/−^, IRF-1^−/−^3^−/−^, IRF-1^−/−^7^−/−^ and IRF-1^−/−^3^−/−^7^−/−^ M-CSF bone marrow derived macrophages were infected with MVA or MVAΔF1L at an M.O.I of 10 for 20 h. Cell death was assessed by PI staining and relative numbers were calculated by calculating the % increase in PI proportional to the untreated sample as in Figure 4. (E) T-REx 293 cells stably carrying a κB promoter luciferase reporter were infected with MVA or MVAΔF1L at an M.O.I of 10 for 8 h. 4 hp.i cells were treated with IL-1β (10 ng/ml). Luciferase activity was determined and the index of induction was calculated from the ratio of luciferase activity in treated cells divided by luciferase activity in untreated cells. (* indicates significance according to the student's t-test, p≤0.05 with data showing mean/SEM of n≥3).

These data suggested that the pathway to apoptosis induction involved the signalling axis from cytosolic helicases, their mitochondrial adapter MAVS and the transcription factor IRF3. MEFs from mice lacking MAVS were significantly protected against MVAΔF1L induced apoptosis, consistent with the MAVS-dependent induction of Noxa-promoter activity observed in the experiments described above (Figure 6A). Mouse Noxa protein is difficult to detect with the antibodies available. We were able to detect Noxa-induction upon infection with MVA in the presence of the proteasome inhibitor MG-132. No Noxa was detectable upon infection of MAVS-deficient MEFs (Figure 6B). A MAVS-dependent signal is thus induced upon infection with MVA orMVAΔF1L, which causes the IRF-3-dependent up-regulation of Noxa and apoptosis.

One of the primary functions of this pathway is the induction of type 1 interferons [27]. INF-β secretion can be detected in the supernatant of MVA or MVAΔF1L infected macrophages (Supplementary Figure S8B) (this has been reported for MVA previously [28], [29]. IFN-β often serves to amplify an IRF-dependent response. We therefore tested the effect of IFNβ signalling on apoptosis induced by MVA or MVAΔF1L. INFβ-secretion was induced to a similar extent by infection with MVA or MVAΔF1L (Supplementary Figure S8B). When IFN-β-signalling was disrupted by the use of macrophages from mice deficient in the IFN I receptor (IFNAR) there was again protection against apoptosis induced by viral infection to a similar extent as by the loss of Noxa or IRF-3 (Figure 6C and D).

However, disruption of IFN-β-signalling failed to inhibit IRF-3 translocation to the nucleus in response to MVA infection in HeLa cells (transfected with siRNA against IFNAR) or macrophages (from IFNAR-deficient mice) (Supplementary Figure S9A, S9D and S9E). IRF-3 translocation to the nucleus was slightly slower in HeLa cells transfected with IFNAR-specific siRNA (Supplementary Figure S9A, and S9B), concurrent with reduced apoptosis of HeLa cells treated with siRNA to IFNAR (Supplementary Figure S9C). No differences were detectable by analysis of IFNAR-translocation by Western blot of cytosolic and nuclear fractions in either cell type (Supplementary Figure S9D, E). The activation of IRF-3 is therefore probably insufficient for induction of Noxa; this latter step likely requires IFN-signalling through Stat proteins.

NF-κB, which is activated by MAVS and can contribute to IFN I-signalling did not appear to play a role in the induction of Noxa as no NF-κB activity was induced by viral infection (Figure 6E). Viral infection also failed to increase the NF-κB response to extrinsically added IL-1 (there was even some reduction, which may be due to the expression of a functional soluble IL-1-receptor by MVA [30], Figure 6E).

**Figure 7:**
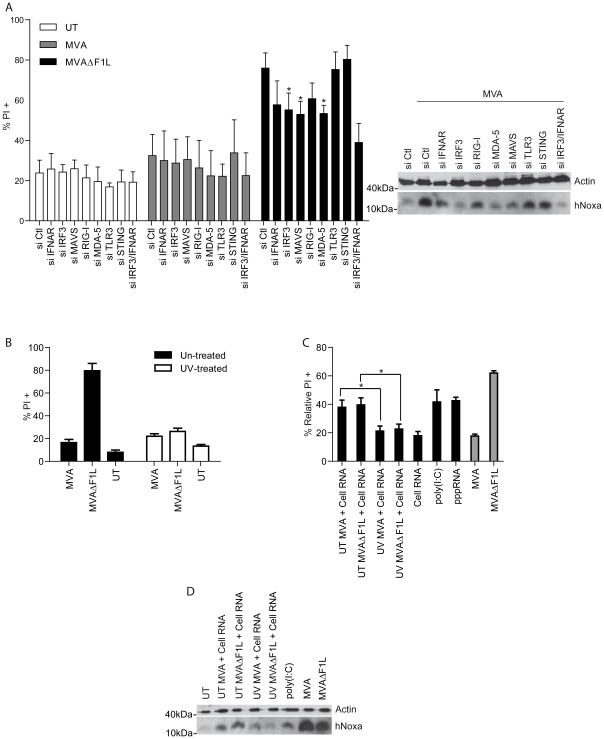
MVA-derived RNA is capable of inducing Noxa and apoptosis. (A) HeLa cells were transfected with the indicated siRNA 24 h prior to infection with MVA or MVAΔF1L at an M.O.I of 10 for 20 h. Cell death was assessed by PI staining. MVA infected HeLa cells were further assessed for Noxa levels by Western blotting. (B) MVA or MVAΔF1L (3×10^6^ IU) were resuspended in 1 ml medium and treated with 1 µg/ml of psoralen (4 -aminomethyl-trioxsalen) and then irradiated with UV light for 15 min. Untreated or treated MVA or MVAΔF1L was then used to infect HeLa cells at an M.O.I of 10 for 20 h. Cell death was assessed by PI staining. (C) Black Bars: total RNA from HeLa cells infected with UV treated or untreated (UT) MVA or MVAΔF1L (or uninfected HeLa cells) (2 µg), poly (I∶C) (10 µg) or pppRNA (1 µg) was transfected using Fugene reagent into HeLa cells. Cell death was assessed 24 h later by PI staining and relative numbers were calculated by calculating the % increase in PI proportional to the Fugene-alone treated sample with the following equation: (((% RNA+Fugene Treated PI^+^)−(% Fugene Treated PI^+^))/(100−% Fugene Treated PI^+^)*100). Grey bars: HeLa cells were infected with MVA or MVAΔF1L at an M.O.I of 10 for 20 h. Cell death was assessed by PI staining and relative numbers were calculated by calculating the % increase in PI proportional to the untreated sample as in Figure 4. (D) HeLa cells from C were further assessed for Noxa levels by Western blotting. (* indicates significance according to the student's t-test, p≤0.05 with data showing mean/SEM of n≥3).

These results show that MVA- and MVAΔF1L-infection induces apoptosis dependent on the signalling adapter MAVS, the transcription factor IRF-3, IFN-β and the BH3-only protein Noxa. We then turned to HeLa cells, on one hand to confirm these results in another cell type and on the other hand to test the role of the helicases upstream of MAVS by RNAi. The individual genes were knocked down by transfection of siRNA, and the cells were infected with MVA or MVAΔF1L. No apoptosis induction by MVA was seen as expected (Figure 7A). RNAi directed against IFNAR, IRF3 or MAVS all substantially reduced apoptosis induced by MVAΔF1L as well as the induction of Noxa by MVA (Figure 7A). Mixed RNAi against both IRF3 and IFNAR showed a slightly enhanced effect. Recognition and apoptosis pathways therefore appear to be similar in mouse and human cells.

MAVS is the signalling adapter of two helicases, RIG-I and MDA5, which recognise viral RNA-species [27]. This signalling system has been very well characterised for RNA-viruses and although it is generally assumed that similar receptors exist for viral DNA the only receptor universally accepted drives the activation of the inflammasome rather than of the type 1 interferon signalling [31].

However, viral RNA produced during infection with a DNA-virus like MVA may still be recognised by the helicase machinery, and evidence has recently been reported that MVA can be recognised by MDA5 [28]. It has also been described that MVA induces the formation of viral dsRNA in the cytosol post RNA secretion from the viral particle [32]. Indeed, RNAi against MDA5 and to a slightly lesser extent against RIG-I provided protection against apoptosis induced by MVAΔF1L, concomitant with the diminished induction of Noxa protein (Figure 7A).

This suggested that indeed viral RNA, generated during MVA infection, is recognised by these helicases to signal through MAVS for the induction of Noxa. Although we saw no protection by transfection of a RIG-I helicase fragment (RIG-IC; constructed as a dominant negative inhibitor of RIG-I-signalling [33]), the inhibitory helicase-like molecule LGP2 [34] reduced apoptosis induced by MVAΔF1L (Supplementary Figure S10A). More importantly, overexpression of LGP2 but not RIG-I or RIG-IC led to an increase of FLAG expressing attached (viable) cells after infection with MVAΔF1L indicating protection of transfected cells against apoptosis induction by MVAΔF1L (Supplementary Figure S10B). This suggested that it is the viral RNA produced during infection with MVA that is recognised and that triggers the activation of the apoptotic pathway.

To test this hypothesis we performed experiments with virus treated with UV-light. Complete cross-linking of the viral genome by UV-treatment results in VACV or MVA that can still infect cells but cannot efficiently generate viral RNA [35]. UV-treated MVA or MVAΔF1L failed to induce apoptosis in HeLa cells (Figure 7B). Lack of apoptosis by UV-treated MVAΔF1L correlated with lack of Noxa induction (data not shown). We then infected cells with MVA or MVAΔF1L and isolated total RNA; this RNA contains host cell and viral RNA in the case of untreated virus but only host cell RNA when isolated from cells that remained un-infected or had been infected with UV-treated virus. When this RNA was transfected into uninfected HeLa cells, it induced substantial apoptosis (to about the same extent as transfected poly I∶C or 3′-triphosphate RNA) while RNA extracted from cells infected with UV-treated virus (containing no viral RNA) had no pro-apoptotic effect (Figure 7C).

Viral RNA mediated induction of apoptosis correlated with the induction of Noxa protein (Figure 7D) and was inhibited in HeLa cells carrying Noxa-specific shRNA (Supplementary Figure S11B).HeLa cells transfected with siRNAspecific for MAVS, IRF-3, MDA-5, or IFNAR also showed decreased apoptosis when transfected with RNA from infected cells (Supplementary Figure S11B). This supports the hypothesis that viral RNA, produced during infection with MVA, is the active component in terms of apoptosis induction upon virus recognition.

## Discussion

In this study we provide evidence that MVA triggers apoptosis in host cells upon the recognition of viral RNA by host cell helicases. This signalling utilises the known pathway encompassing cytosolic helicases, the mitochondrial adapter MAVS, the type 1 interferon signalling machinery and the induction of the BH3-only protein Noxa. Bim makes a small contribution, and F1L can inhibit apoptosis pathways triggered by MVA.

How Bcl-2-family members interact to induce or to inhibit apoptosis is still not entirely clear. Bim-induced apoptosis is often linked to transcriptional induction of Bim, especially in the immune system [4]. There was no induction but a small decrease of Bim protein during MVA-infection. The role of Bim may therefore in this case lie more in the steady state inhibition of anti-apoptotic Bcl-2 proteins, which may lower the general threshold of apoptosis induction and account for the relatively mild protective effect of Bim-loss on apoptosis induction by MVAΔF1L. This interpretation is also in accordance with our finding that F1L (as expressed during infection) provides very little protection against direct expression of Bim or the effect of the Bim BH3-peptide.

Other BH3-only proteins did not seem to make major contributions, as suggested by the lack of protection by their individual loss and by the strong protection by combined loss of Bim and Noxa. This protection was however incomplete while the combined loss of Bax and Bak provided complete protection. Therefore, other BH3-only proteins may make minor contributions that are too small to be apparent when the BH3-only protein is deleted on its own. Alternatively, there may be BH3-only independent activation of Bax/Bak. How this could occur is unclear but it has been demonstrated for heat shock [36], and it is conceivable that viral infection causes a sufficiently strong disturbance of cellular systems to bring about such a (still relatively minor) effect.

Upon infection of mice MVA preferentially infects macrophages and DC although T and B cells are also infected. Lymphocyte apoptosis is to a large extent regulated by Bim [37] and it is therefore not surprising that loss of Bim had the strongest protective effect in lymphocytes. The combined role of Bim and Noxa was apparent in all cell types tested. The main difference was that MVA induced substantial apoptosis in macrophages and DC but little in lymphocytes and none in fibroblasts and epithelial cells. The simplest explanation for this would be different expression levels of pro-apoptotic proteins in different cell types.

It has to be pointed out that it is still uncertain how F1L functions molecularly. However relevant the detectable binding to Bak and to Bim may be (shown for VACV F1L), this is not sufficient to explain the complete protection observed during infection. It has recently been suggested that VACV F1L acts like Mcl-1 [38]. Although this may be the case in the circumstance of viral infection, Mcl-1 has a much broader activity in terms of binding specificity for pro-apoptotic Bcl-2 family proteins.

Noxa is required for full apoptosis in cells infected with MVAΔF1L, and F1L therefore very likely interferes with Noxa-dependent apoptosis. Since the Noxa BH3-domain does not bind to F1L with significant avidity [10] it seems unlikely that F1L binds Noxa directly. Noxa may inactivate Mcl-1, releasing Bak, which is then inhibited by F1L. An additional function of F1L has recently been reported, namely the direct inhibition of caspase-9 [12], and this mechanism may add to the anti-apoptotic effect of MVA-infection.

The earliest components of the apoptotic pathway are therefore the BH3-only proteins Bim and Noxa. The activity of Noxa is linked to its transcriptional induction [39]. Although viral replication blocks cellular transcription, the infected cell still appears to have the time to induce Noxa protein. The induction of Noxa follows a pathway that, with some variations, has been described in other situations. RIG-I/MDA5 signal through MAVS to activate IRFs, which induce IFN-β [27]. IRF-3 may directly induce Noxa-expression, and the amplifying effect of IFN-β may be through increased IRF-3 expression [28]. Alternatively, IFN-β may induce Noxa; this is suggested by the requirement of IFN I-signalling for full apoptosis induction in cells infected with MVAΔF1L. In melanoma cells, the activation of RIG-I or MDA5 by transfection of 3′-triphosphate RNA or poly I∶C, respectively, caused Noxa-induction and Noxa-dependent apoptosis without additional type 1 interferon signalling [40] while during MVA-infection this feedback, probably signalling through Stat-proteins, appears to be necessary.

Not only (RNA-recognising) helicases can induce apoptosis but also TLR3 (using a mitochondria-independent pathway) [41], which also recognises viral (double-stranded) RNA. Intriguingly, the DNA-recognising TLR, TLR9 does not have this potential (nor do the RNA-receptors TLR7/8). It is intriguing to observe that even a DNA virus is recognised by RNA-sensors to induce apoptosis. It has been demonstrated recently that MVA-infection of macrophages leads to MDA5-dependent production of IFN-β while RNAi to RIG-I had no effect on IFN-β-production [28]. Our results suggest that RIG-I also makes some contribution; perhaps there is some cell-to-cell variation, or a different time course of involvement of the two helicases that makes apoptosis-induction more dependent on RIG-I than IFN-β-induction.

Recent work shows that RIG-I can recognise RNA transcribed from DNA by RNA polymerase III [42], [43], and such a mechanism could operate for DNA-viruses. MDA5 probably mainly recognises double-stranded RNA [44], and the recognition by MDA5 may be limited to some DNA-viruses that, like Poxviruses, generate dsRNA-intermediates due to overlapping reading frames [32]. But since all viruses at some stage will synthesise RNA, this is clearly the more general infection-associated molecule than DNA, and to focus on RNA as recognition molecule for viruses makes sense.

Apoptosis is one of the defence mechanisms against viruses. Viruses pre-date the evolution of metazoan apoptosis, and it is certainly conceivable that viral infection was one of the driving forces in the evolution of the apoptotic apparatus. As an essentially cell-autonomous defence system, apoptosis does not require cellular specialisation. In a complex organism like the human, apoptosis is very likely one of several defence mechanisms but may well contribute to host defence against microbial infection.

## Materials and Methods

### Ethics statement

Animal experiments were conducted according to the legal framework in Germany (as set by federal law in the ‘German Animal Protection Act’ (Tierschutzgesetz)) and had been approved by the regional authorities (Regierung von Oberbayern; permit number:211-2531-6-8/99) following the regular procedures of application and approval.

### Cells and viruses

wt (3T3), wt(SV40) , Bak^−/−^(SV40), Bax^−/−^(SV40), Bak^−/−^Bax^−/−^(SV40), Bim^−/−^(3T3), Puma^−/−^(SV40), Noxa^−/−^(3T3), Bid^−/−^(3T3), Bad^−/−^(3T3), PKR^−/−^ (3T3), IRF-1^−/−^ (3T3), IRF-3^−/−^7^−/−^ (3T3), MAVS^−/−^ (SV40), MEFs were grown in Dulbecco's Modified Eagle Medium (DMEM), supplemented with 10% fetal calf serum, 1% Penicillin /Streptomycin, and 55 µM 2-mercaptoethanol. (MAVS^−/−^ were kindly provided by Zhijian J. Chen, Dallas).HeLa cells were grown in DMEM, 10%FCS, 1%Penicillin /Streptomycin. wt TetBim_s_ MEFs and T-REx 293 cells stably carrying a κB promoter luciferase reporter were cultured in DMEM, 10%FCS (tetracycline-negative; PPA laboratories), 1%Penicillin /Streptomycin supplemented with 5 µg/ml blasticidin and 125 µg/ml zeocin. Macrophages and Dendritic Cells were differentiated (7 days) and maintained in RPMI, 10%FCS, 1%Penicillin /Streptomycin, 55 µM 2-mercaptoethanol, supplemented with 10% M-CSF or GM-CSF enriched medium obtained from the supernatant of LCCM Hybridomas or B16 cells in the same medium, respectively. T-cells were cultured and activated in RPMI, 10%FCS, 1%Penicillin/Streptomycin, 55 µM 2-mercaptoethanol, supplemented with 1% glutamine and Con A (2 µg/ml; Amersham Pharmacia Bioscience). B cells were cultured in OPTI-MEM I+GlutaMAX-I, 10%FCS, 1%Penicillin /Streptomycin, 55 µM 2-mercaptoethanol. ER-Hox B8 wt and IFNAR^−/−^ cells were cultured in RPMI, 10% FCS, 1%Penicillin /Streptomycin, 55 µM 2-mercaptoethanol, supplemented with 1% GM-CSF enriched medium and 1 µM βestridiol (Sigma). Differentiation was achieved by washing cells and culturing them in RPMI, 10%FCS, 1%Penicillin /Streptomycin, 55 µM 2-mercaptoethanol, supplemented with 10% GM-CSF enriched medium.

shHela cells were generated by retroviral infection with lentivirus driving shRNA against luciferase (5′-GUGCGCUGCUGGUGCCAAC-3′) and Noxa (5-GAAGGTGCATTCATGGGTG-3) in a GFP expressing lentiviral vector pLVTHM. Lentiviralparticles were generated by transfecting 293FT cells together with the packaging vectors pMD2.G and psPAX2. Surviving cells were grown and collected and sorted to produce polyclonal GFP+ populations as reported earlier [45].

Chicken embryonic fibroblasts (CEF) were prepared freshly from 10 day old embryos, cultured in Earl's minimum essential medium and used in the second passage. MVA and MVAΔF1L were routinely propagated and tittered by vaccinia virus-specific immunostaining on CEF. Viruses were purified by ultracentrifugation through sucrose and virus stocks were maintained at −80°C in aliquots containing ≥ 2×10^8^ IU/ml [46].

MVA expressing the enhanced green-fluorescence gene under control of the VV natural early/late promoter P7.5 (MVA-GFP) has been described previously [47]. MVA viruses were propagated and titrated by following standard methodology [46].

### Antibodies for Western blotting

Antibodies used for Western blot were specific for h/mBim, Bad, Puma (Cell Signalling) hNoxa (Alexis), Bid (polyclonal rabbit anti-Bid was kindly provided by David Huang, Melbourne) β-actin, β-tubulin (Sigma), F1L (polyclonal rabbit anti-F1L was kindly provided by Antonio Postigo and Michael Way, London), Bak (NT; Upstate), cytochrome c, hMcl-1 (BD Pharmingen), mNoxa, mIL-18 (Abcam), H3L (Polyclonal antiserum specific for VACV H3 was generated by repeated immunization of a rabbit using a synthetic peptide representing amino acids 247–259 within the H3 protein as a conjugate to keyhole limpet hemocyanin described by others [48]), GAPDH (Millipore), IRF3 (Santa Cruz Biotechnology) Caspase-8 (Cell Signalling), Bak (4B5, kindly provided by Ruth Kluck, Melbourne).

### Cellular preparation

Spleen single cell suspensions from wt, Bim^−/−^, Noxa^−/−^, and Bim^−/−^Noxa^−/−^ mice (all C57BL/6 background) were resuspended in Amonium chloride lysis buffer (150 µM NH_4_Cl, 10 mM NaHCO_3_, 1 mM Na_2_EDTA pH = 7,4 ) for 5 min and then washed in PBS. Cells were then further resuspendedin sorting buffer (PBS, 0.5%BSA), washed and were labelled with anti-B220-FITC (BD Pharmingen), FITC-anti-NK 1.1 (BD Pharmingen), and anti-MHCII-FITC (BD Pharmingen) antibodies for 30 min at 4°C. Labelled cells were washed and resuspended with anti-FITC micro beads (MiltenyiBiotec, Auburn, CA) for 30 min at 4°C. Labelled cells were removed on MACS columns (MiltenyiBiotec, Auburn, CA) in order to obtain T cell populations. To check purity cells were stained with CD3-APC antibody (Purity ≥ 90%). Cells were activated with ConA (2 µg/ml) for 3 days and split for infection.

Splenocytes from mice of the genotypes above were resuspended in PBS, washed and were labelled with anti-B220 micro beads (MiltenyiBiotec, Auburn, CA) for 30 min at 4°C or with FITC-anti-B220 and treated as above. Labelled cells were purified on MACS columns (MiltenyiBiotec, Auburn, CA). Dendritic cells from wt, Bim^−/−^, Noxa^−/−^, Bim^−/−^Noxa^−/−^, IRF-1^−/−^, IRF1^−/−^3^−/−^, IRF-1^−/−^7^−/−^, IRF-1^−/−^3^−/−^7^−/−^ and IFNAR^−/−^ mice (all C57BL/6 background) were derived from bone marrow single cell suspensions cultured with enriched GM-CSF medium for 7 days. On day 1 after isolation cells were passaged to separate them from attached fibroblasts. Medium was further enriched with GM-CSF on day 3. Floating cells were collected and split for treatment on day 7. Macrophages were isolated in the same way but with enriched M-CSF and attached cells were used for infection on day 7.

Bim^−/−^, Noxa^−/−^, Bim^−/−^Noxa^−/−^ mice were kindly provided by Claire Scott, Philippe Bouillet or Andreas Strasser, Melbourne. p53^−/−^ mice were kindly provided by David Huang, Melbourne. IRF-1^−/−^, IRF-1^−/−^3^−/−^, IRF-1^−/−^7^−/−^ mice were kindly provided by Hermann Wagner, Munich. IFNAR^−/−^ and IRF-1^−/−^3^−/−^7^−/−^ mice were kindly provided by AdmarVerschoor, Munich.

### Death assays and treatments

Cell death was induced by treatment with MVA or MVAΔF1L (M.O.I  =  10) in DMEM medium 2%FCS enriched to 10% FCS 8 h.p.i for 20 h, ultra violet radiation (UV,100 J/m^2^) 20 h, etoposide (10 µM, Sigma) 20 h, or staurosporine (1 µM) 8 h. In the indicated experiments cells were exposed to MG-132 (40 µM) for 4 h, 16 h.p.i. Floating cells and trypsinised attached cells were combined and incubated in PBS containing 50 µg/ml propidium iodide (PI) for 5 min on ice before measuring PI uptake by flow cytometry.

Alternatively floating cells and trypsinised attached cells were combined and incubated in 3.7% paraformaldehyde for 20 min at room temperature, washed three times in PBS and incubated in mild-permeabilisation buffer (PBS, 3% FCS, 0.5% Saponin) for 20 min. Cells were stained with primary monoclonal active caspase 3 antibody (BD Pharmingen) and with FITC- goat anti rabbit secondary antibody (Dianova) and measured for FITC+ cells by flow cytometry.

### Cellular fractionation for cytochrome c release assay by Bim BH3 peptide or IRF3 translocation

Cells were harvested, washed in ice cold PBS, and resuspended at 1×10^7^cells/ml in permeabilisation buffer (20 mM HEPES/KOH pH 7.5, 100 mM sucrose, 2.5 mM MgCl_2_, 100 mMKCl, 0.025% digitonin, 1× complete protease inhibitors (Roche)). Cell membrane permeabilisation was verified by uptake of trypan blue after incubation on ice for 10 min. Membrane (pellet) and cytosolic (cytosol) fractions were separated by centrifugation at 13,000× g for 5 min.

In the case of cytochrome *c* release assays the pellet fraction was resuspended in digitonin-free permeabilisation buffer and treated with Bim BH3 peptide (MRPEIWIAQELRRIGDEFNA ; Biosynthan GmbH; HPLC cleanness >90%) for 30 min at 30°C. Supernatant and membrane (pellet) fractions were separated by centrifugation (13,000× g for 5 min). Cytosol, supernatant and pellet fractions were assessed by Western Blot for cytochrome *c*, Bak as a membrane control and F1L as an infection control.

In the case of IRF3 translocation assayspellet fractions were resuspended in RIPA buffer (Sigma) wit 1× complete protease inhibitors and prepared along side the cytosol fractions for Western blotting.

### Induction of apoptosis by tetracycline-induced expression of Bim_S_

MEFs carrying the tetracycline-repressor were generated as described for HeLa cells [49].

Cells were infected with MVA or MVAΔF1L for 8 h prior to addition of tetracycline (0.1 µg/ml) for further 12 h.

### MVA-GFP infection of mice

Mice (n  =  3) were infected i.v. with 3×10^8^ IU of MVA-GFP for 3 hours before spleens were harvested. Splenocytes were stained with the following antibodies for phenotypical analysis: CD11c APC (HL3), CD3 PB(500A2), Gr-1 PE (1A8; all from BD Pharmingen), CD11b PerCP-C5.5 (M1/70), MHCII eF450 (M5/114.15.2) and B220 PE (RA3-6B2; all eBioscience).Fluorochrome-conjugated isotype-matched monoclonal antibodies were used as controls. Anti- CD16/CD32-Fc-Block (BD Biosciences) was included. Propidium iodide (Molecular Probes) was added immediately before analysis for live/dead discrimination. Data were acquired by FACS analysis on a FACSCanto (BD Biosciences) and analyzed with FlowJo software (Tree Star, Inc.). Macrophages (MHC II high /CD11c intermediate / CD11b high), dendritic cells (MHC II high /CD11c high), B cells (MHC II high-intermediate /CD45R (B220) high), Granuloytes (Gr-1 high / CD11b high) an T cells (CD3+) were identified within GFP+ splenocytes. Similarly, three mice were simultaneously infected i.v. with 3×10^9^ IU of MVA-GFP for 3 hours before spleens were harvested. Splenocyte preparations were gated for GFP^+^ cells. Splenocytes were further assessed for the indicated phenotypical markers. Macrophages (CD11b^+^/ Gr1^−^; eBioscience/ BD Pharmingen) DC (CD11c; BD Pharmingen) T cells (CD3; BD Pharmingen), B cells (CD19; BD Pharmingen) and neutrophil granulocytes (CD11b+/Gr-1+).

### Noxa promoter induction

MEFs (wt, IRF1^−/−^, IRF3^−/−^7^−/−^, and MAVS^−/−^) were seeded at 50,000 cells/well in a six well plate. 24 h later cells were transfected using Fugene liposomes (Roche) with different reporter constructs of the Noxa promoter (pGL3-basic plasmid carrying wt or a double mutant Noxa 1.2 kb promoter region[24]) or pEGFP-C1 as a transfection control (constructs were kindly provided by Christophe Lallemand, Villejuif). Twenty-four hours later transfected cells were harvested and in cases when the pEGFP controls were effectively transfected to values above 20% (EGFP positive cells were determined by flow cytometry), were split in 96 well plates at 5,000 cells per well. 16 h post seeding cells were infected with MVA or MVAΔF1L for further 8 h. Cells were washed, lysed in lysis buffer (Promega), and incubated while gently shaking at 37°C for 30 min. Luciferase activity was detected using Orion MicroplateLuminator (Berthold Detection Systems) and an luciferase detection buffer (Promega).

### IFN-β production

Wt macrophages were seeded at 300,000 cells/well. Twenty-four hours post seeding cells were infected with MVA or MVAΔF1L for 8 h. Supernatant was collected and stored at −80°C. IFN-β was detected using INF-β capture antibody (US Biological) and anti-mouse INF-β primary detection antibody (TEBU BIO).

### NFκB activity detection

T-REx 293 cells stably carrying a κB promoter luciferase reporter were seeded at 20,000 cells/well in a 96 well plate. Twenty-four hours post seeding cells were infected with MVA or MVAΔF1L for 8 h and treated with recombinant human IL-1β (Peprotech) 10 ng/ml for a further 4 h. Luciferase activity was detected as above.

### siRNA transfection of HeLa cells

HeLa cells were seeded at 80,000 cells per well and 24 h later transfected with LipofectamineRNAiMAX (Invitrogen) as instructed by the manufacturer. A total of 30 pmoles of siRNA directed against IFNAR (AACAGCCAUUGAAGAAUCUUC) [50], IRF3 (AACCGCAAAGAAGGGUUGCGU) [50], MAVS (CCACCUUGAUGCCUGUGAA), RIG-I (GUAUCGUGUUAUUGGAUUA), MDA-5 (AUCACGGAUUAGCGACAAA), TLR3 (CAGCATCTGTCTTTAATAA) or STING (GCATCAAGGATCGGGTTTA) were used in each transfection (these sequences were kindly provided by Robert Besch, Munich). Twenty-four after transfection cells were infected with MVA or MVAΔF1L for 20 h.

### Immunofluorescence microscopy

HeLa cells cells were seeded at 80,000 cells per cover slip containing well and 24 h later treated with siRNA as above. ER-HoxB8 macrophages were seeded at 150,000 cells percover slip containing well. MVA infection was carried out as above for the desired times of infection. Cells were washed and fixed in 3.7% formaldehyde prior to permeabilizing with 0.2% Triton-X in PBS. Cells were washed, blocked in 5%BSA PBS and incubated for 45 min in blocking buffer with a 1∶100 dilution of anti-IRF3 (Santa Cruz Biotechnology). Cells were further washed and incubated in blocking buffer with a 1∶500 dilution of dyelight conjugated anti-rabbit (Dianova) antibody and 1∶15000 dilution of Hoechst dye (Sigma) for 45 min. Samples were further washed and slides were mounted. Images were taken with a Zeiss Axioplan 2 Imaging microscope using the 40× magnification lens for all images.

### MVA UV treatment

MVA or MVAΔF1L (3×10^6^ IU) were resuspended in 1 ml DMEM containing 2%FCS and treated with 1 µg/ml of psoralen (4 -aminomethyl-trioxsalen) and then irradiated with UV for 15 min in a Stratalinker 1800 UV crosslinking unit (Stratagene, La Jolla, CA, USA).

### MVA total RNA extraction

1×10^6^ uninfected or MVA or MVAΔF1L infected cells (4 hp.i) were trypsinized and collected for total RNA purification. Isolation of total RNA was done with the RNeasy Mini Kit (Qiagen) using QIAshredder (Qiagen) for lysate homogenization as stated in the manufacturer's instructions. RNA concentrations collected were in the range of 150–250 ng/µl and had A_260_∶A_280_ values of 1.8–2.2. Total RNA was stored at −80°C until used for transfections. Total RNA was transfected into HeLa cells with Fugene at a ratio of 2 µg RNA and 8 µl Fugene. HeLa cells were simultaneously transfected with pppRNA (1 µg∶8 µl Fugene) or poly(I∶C) (10 µg∶8 µl Fugene).

### RIG-I and MDA-5 inhibition assays

HeLa cells were transfected with pEF-BOS plasmids containing FLAG-tagged RIG-I, RIG-I helicase fragment RIG-IC, LGP2 or pEGFP (transfection control) using Fugene according to the manufacturers protocol (RIG-I plasmids we kindly provided by Katharina Eisenächer and Anne Krug, Munich). The ratio Fugene(µl)∶DNA(µg) was optimized to 8∶2.Transfected cells were infected with MVAΔF1L for 20 h. Cells were assessed for death or for FLAG expression. Cells were fixed in 3.7% paraformaldehyde for 20 min at room temperature, washed three times in PBS and incubated in mild-permeabilisation buffer (PBS, 3% FCS, 0.5% saponin) for 30 min. Cells were stained with anti-FLAG M2 mouse monoclonal antibody (Sigma) and goat anti-mouse Alexa 488 labeled secondary antibody (Molecular Probes) and analyzed by flow cytometry.

## Supporting Information

Figure S1 Time course of F1L expression. (A) MEFs were infected with MVA or MVAΔF1L at an M.O.I of 10 for 20 h and the levels of H3L (an MVA membrane protein) and F1L were assessed by WB. (B) MEFs were treated as in (A) and the levels of F1L were assessed at the indicated time points by WB.
(PDF)

Figure S2 MVAΔF1L induced apoptosis is predominantly mediated by Noxa. (A) MEFs were infected with MVA or MVAΔF1L at an M.O.I of 10. Apoptosis was assessed at the indicated times by PI staining. (B) Samples from Figure 1 (A) were assessed for cytosolic active caspase-3 20 h.p.i. (C) Samples from Figure 1 (B,C) were assessed for cytosolic active caspase-3. (D) Samples from Figure 1 (D) were assessed for apoptosis at time point 20 h.p.i. by PI staining and for cytosolic active caspase-3. Western blot inserts represent controls for absence of protein expression in MEFs. (* indicates statistical significance according to the student's t-test, p≤0.05 with data showing mean/SEM of n≥3).
(PDF)

Figure S3 Apoptosis caused by tetracycline inducible Bims expression in MEFs is only mildly reduced by prior MVA-infection. (A) wt Tet Bim_s_ MVA infected or un-infected (at an M.O.I. of 10 for 8 h) were treated with the indicated concentrations of tetracycline for 12 h. Cell death was assessed by PI staining. Black box represents the concentration used for (C). (B) Bim_s_ levels from wt samples from (A) as detected by Western blotting. (C) wt Tet Bims MEFs were infected with MVA or MVAΔF1L at an M.O.I. of 10 for 8 h or left uninfected and were treated with tetracycline (0.1 µg/ml) for 12 h. Apoptosis was assessed by detection of cytosolic active caspase-3 (data are mean/SEM of n≥3).
(PDF)

Figure S4 Infection pattern in primary cells upon infection of mice with high-dose MVA. Three mice were in one experiment infected i.v. with 3×10^9^ IU of MVA-GFP 3 h before spleens were harvested. Splenocyte preparations were stained for the markers shown and gated for GFP^+^ cells for analysis. MVA preferentially infects dendritic cells (CD11c+, second panel from top) and macrophages (CD11b^+^/Gr1^−^, bottom). Infection rates of T cells, B cells and granulocytes (CD3, CD19 and CD11b+/Gr-1+) were lower. Each column shows data from one mouse.
(PDF)

Figure S5 While MVA induced apoptosis is predominantly induced by Noxa, Bim does synergize with Noxa to induce apoptosis in the primary infection target cells of MVA. (A)Samples from Figure 4 were assessed for cytosolic active caspase-3. Active caspase-3 relative numbers were calculated by calculating the % increase in active caspase-3 positive cells proportional to the untreated sample with the following equation: (((% Treated active caspase 3^+^)−(%untreated active caspase 3^+^))/(100−% untreated active caspase 3^+^))*100). (* indicates statistical significance according to the student's t-test, p≤0.05 n≥3). (B) B220^+^ MACS sorted B-cells and NK1.1^−^/B220^−^/MHCII^−^ MACS sorted T-cells were infected with MVA or MVAΔF1L at an M.O.I of 10 for 48 h. Apoptosis was assessed by PI staining and relative numbers were calculated as in Figure 4. (* indicates statistical significance according to the student's t-test, p≤0.05 with data showing mean/SEM of n≥3).
(PDF)

Figure S6 Cell death of hematopoietic cells in culture upon viral infection. (A) The same cellular subsets from Figure 4 were assessed by PI staining at the indicated times with or without infection with MVA or MVAΔF1L (n = 3). (B) Macrophages from Figure 5(A) were assessed by PI staining with or without infection with MVA or MVAΔF1L (n = 3). Values represent raw values obtained from three independent experiments without compensating for the simultaneous death of cells in the untreated samples.
(PDF)

Figure S7 Effect of RNAi to Bim, Noxa and Puma on death during infection of HeLa cells with MVAΔF1L. (A) HeLa cells were infected with MVA or MVAΔF1L at an M.O.I of 10 for the indicated times. Cell death was assessed by PI staining (n≥3). (B) HeLa cells were transfected with the indicated siRNA 24 h prior to infection with MVAΔF1L at an M.O.I of 10 for 20 h. Noxa and Bim levels were assessed before (far right lane) and after infection (all other lanes) (n = 2). (C) Cells from (B) were assessed for apoptosis by detecting cytosolic active caspase-3 (means from n = 2 independent experiments). (* indicates statistical significance according to the student's t-test, p≤0.05 n values indicated).
(PDF)

Figure S8 p53, MyD88 and the inflammasome are not required for Noxa mediated apoptosis even though INF-β is produced. (A) M-CSF bone marrow derived macrophages were infected with MVA or MVAΔF1L at an M.O.I of 10 for 20 h. Cell death was assessed by PI staining (means from two independent experiments) (B) IFN-β concentrations were measured by ELISA in supernatants of wt M-CSF bone marrow derived macrophages uninfected or infected with MVA or MVAΔF1L at an M.O.I of 10 for 8 h (n = 3). (C) wt MEFs were infected with MVA or MVAΔF1L at an M.O.I of 10 for the indicated times. Samples were assessed for IL-18 expression by WB (n = 3). (D) M-CSF bone marrow derived macrophages were treated as in A (n = 3). (* indicates statistical significance according to the student's t-test, p≤0.05 n-values indicated).
(PDF)

Figure S9 IRF3 translocates to the nucleus in response to MVA infection independently of IFNAR. (A) HeLa cells were transfected with the indicated siRNA 24 h prior to infection with MVA or MVAΔF1L (MOI = 10) for the indicated times. Cells were then fixed and stained with antibody against IRF3 and with Hoechst. The images are representative of the general cellular population at the indicated time points post infection (n = 2). (B) Percentage of cells with nuclear IRF3 were calculated by counting cells in 6 different microscopic fields for each time point and dividing the number of cells with nuclear IRF3 by the total number of cells in the field of view. Data are means of two independent experiments (C) siRNA against IFNAR was functional as it was able to inhibit MVAΔF1L induced apoptosis when cells were infected at an MOI of 10 for 20 h. (D) HeLa cells treated as in (A) were collected at the indicated times, digitonin (0.025% w/v) permeabilized and fractionated into cytosolic and total membrane fractions. IRF3 localization was assessed by Western blot. C is the cytosolic fraction released upon cellular membrane permeabilization. P represents the pellet fraction including nucleus, cellular membrane and intact mitochondria. (E) wt and IFNAR^−/−^ ER-HoxB8 immortalized macrophages were infected with MVA or MVAΔF1L at an MOI of 10 for the indicated time points after 7 days differentiation in GM-CSF medium deficient in β-estridiol, and IRF3-translocation was determined as in (D).
(PDF)

Figure S10 LGP2 can act as a dominant negative inhibitor of MVAΔF1L induced apoptosis. (A) pEF-BOS plasmids containing the indicated FLAG-tagged proteins or pEGFP (transfection control) were transfected into HeLa cells. After 24 h cells were infected with MVA or MVAΔF1L at an M.O.I of 10 for 20 h. Cell death was assessed by PI staining and relative numbers were calculated as in Figure 4 (n = 3). (B) Samples from A were stained for intracellular FLAG before and after infection with MVAΔF1L at an M.O.I of 10 for 20 h (n = 2).
(PDF)

Figure S11 Full viral RNA induced apoptosis requires RNA helicase dependent induction of Noxa. (A) HeLa cells were transfected with the indicated siRNA 24 h prior to transfection of total RNA from HeLa cells infected with MVA or uninfected HeLa cells using Fugene reagent. Cell death was assessed 24 h later by PI staining and relative numbers were calculated by calculating the % increase in PI proportional to the Fugene-alone treated sample as in Figure 7 (with data showing mean/SEM of n≥3). (B) Hela cells stably expressing shRNA specific to Luciferase (control) and Noxa were transfected with total RNA from HeLa cells infected with MVA or uninfected HeLa cells using Fugene reagent; or infected with MVA or MVAΔF1L. Cell death was assessed 24 h later by PI staining and relative numbers were calculated by calculating the % increase in PI proportional to the Fugene-alone treated sample as in Figure 7. In the case of infection with MVA or MVAΔF1L relative values were calculated as in Figure 4 (with data showing mean/SEM of n = 3). shRNA efficiency was assessed by Western Blot (right hand panel) (* indicates statistical significance according to the student's t-test, p≤0.05 with data indicating mean/SEM of n≥3; x indicates unspecific band).
(PDF)
