# Supplementary figures and images for: Induction of Noxa-Mediated Apoptosis by Modified Vaccinia Virus Ankara Depends on Viral Recognition by Cytosolic Helicases, Leading to IRF-3/IFN-β-Dependent Induction of Pro-Apoptotic Noxa
Source: PLoS Pathog. 2011 Jun 16;7(6):e1002083. doi: 10.1371/journal.ppat.1002083 (PMC3116819; doi:10.1371/journal.ppat.1002083)

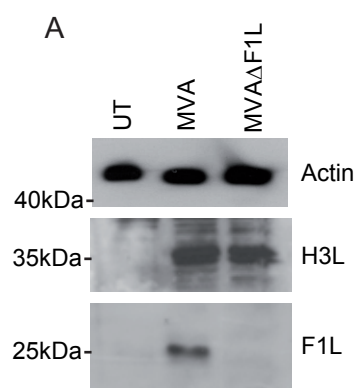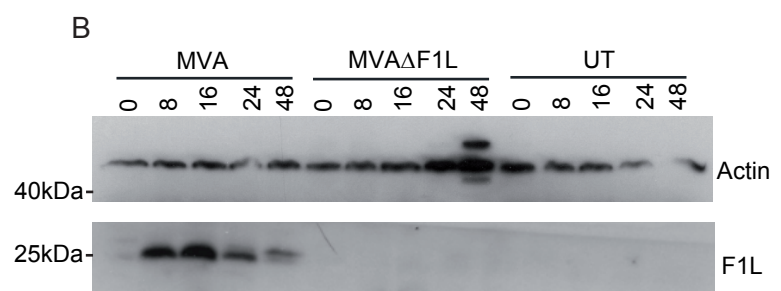

Supplement: Figure S1 — Time course of F1L expression. (A) MEFs were infected with MVA or MVAΔF1L at an M.O.I of 10 for 20 h and the levels of H3L (an MVA membrane protein) and F1L were assessed by WB. (B) MEFs were treated as in (A) and the levels of F1L were assessed at the indicated time points by WB. (PDF) [file ppat.1002083.s001.pdf]

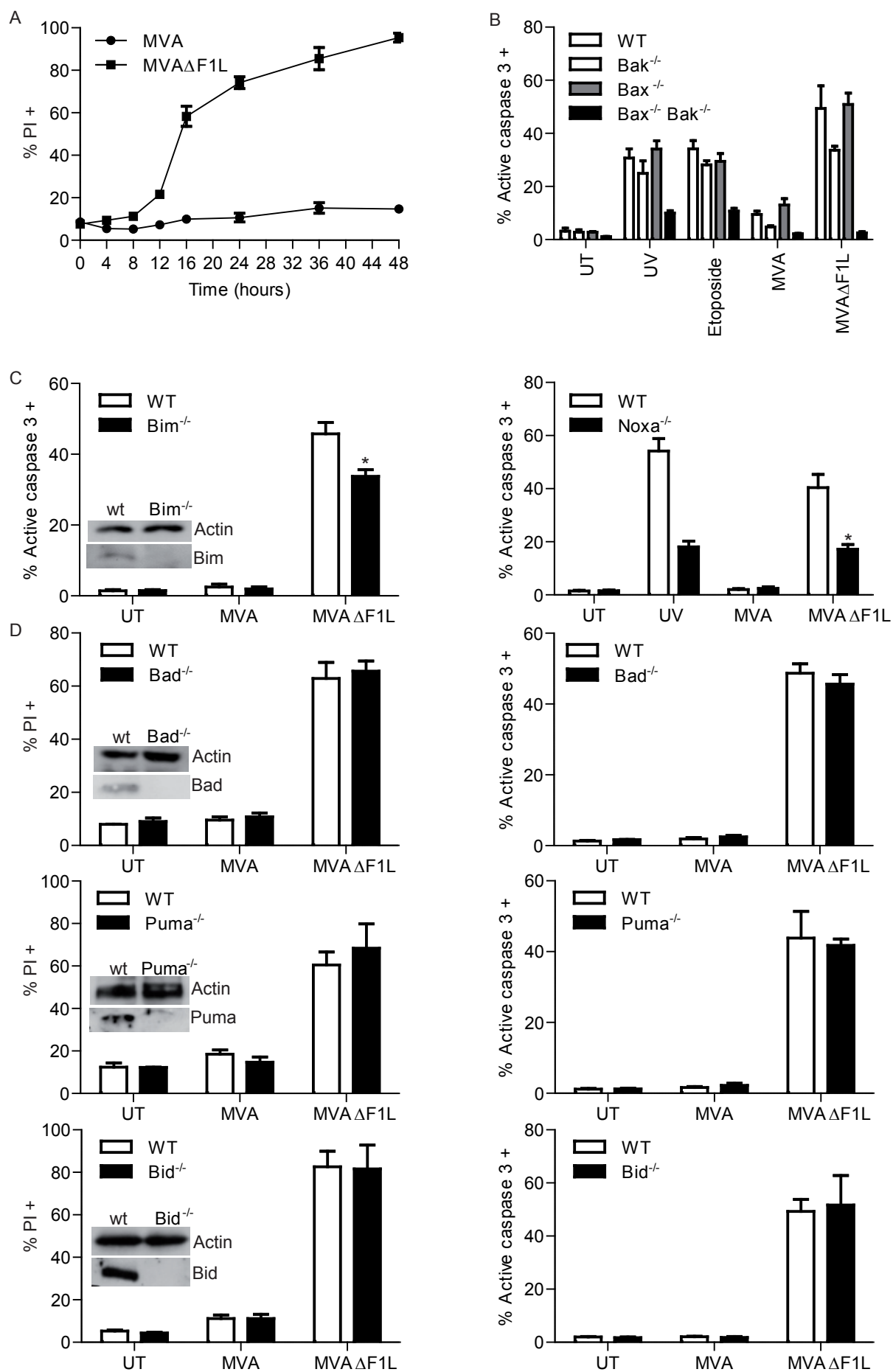

Supplement: Figure S2 — MVAΔF1L induced apoptosis is predominantly mediated by Noxa. (A) MEFs were infected with MVA or MVAΔF1L at an M.O.I of 10. Apoptosis was assessed at the indicated times by PI staining. (B) Samples from Figure 1 (A) were assessed for cytosolic active caspase-3 20 h.p.i. (C) Samples from Figure 1 (B,C) were assessed for cytosolic active caspase-3. (D) Samples from Figure 1 (D) were assessed for apoptosis at time point 20 h.p.i. by PI staining and for cytosolic active caspase-3. Western blot inserts represent controls for absence of protein expression in MEFs. (* indicates statistical significance according to the student's t-test, p≤0.05 with data showing mean/SEM of n≥3). (PDF) [file ppat.1002083.s002.pdf]

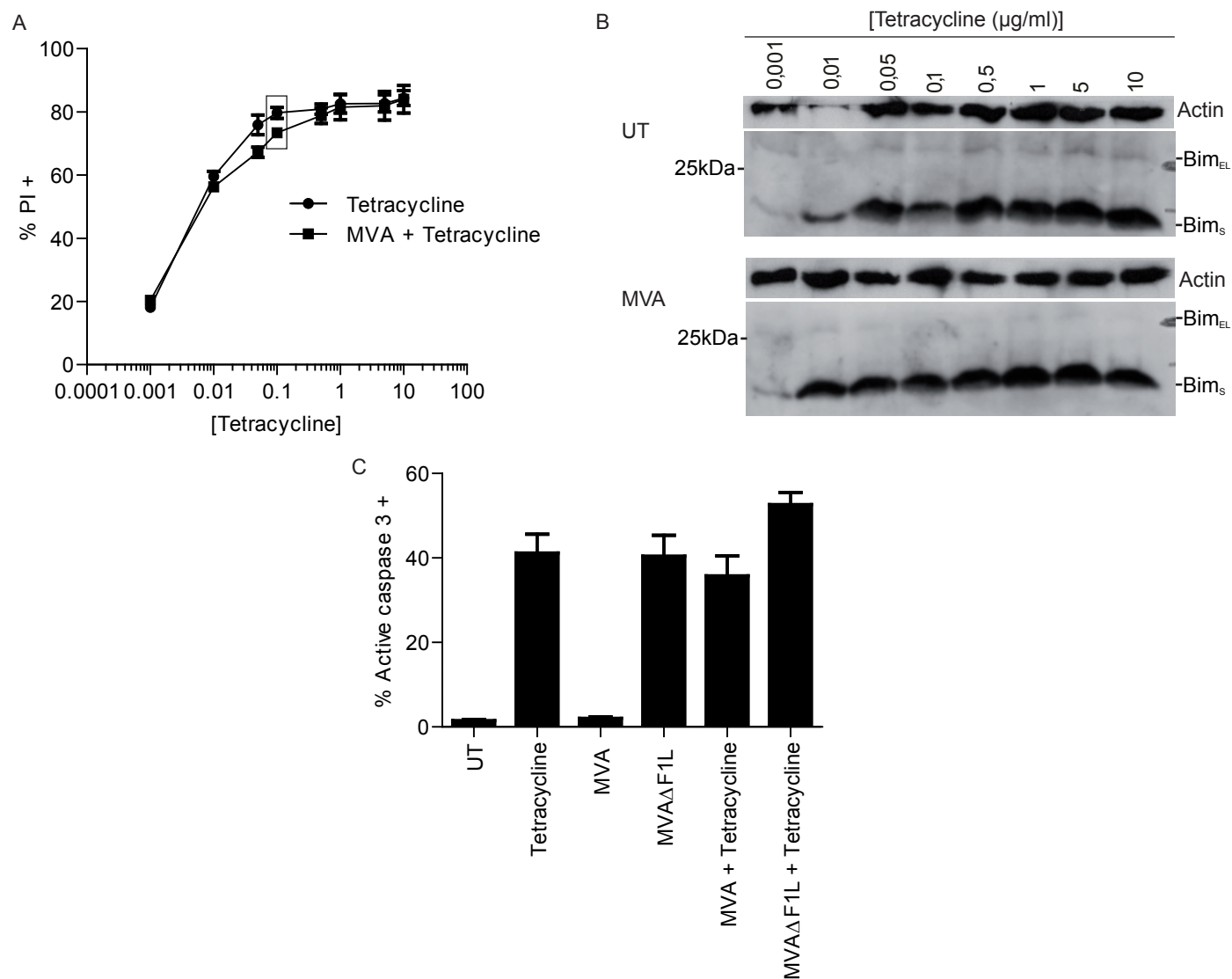

Supplement: Figure S3 — Apoptosis caused by tetracycline inducible Bims expression in MEFs is only mildly reduced by prior MVA-infection. (A) wt Tet Bims MVA infected or un-infected (at an M.O.I. of 10 for 8 h) were treated with the indicated concentrations of tetracycline for 12 h. Cell death was assessed by PI staining. Black box represents the concentration used for (C). (B) Bims levels from wt samples from (A) as detected by Western blotting. (C) wt Tet Bims MEFs were infected with MVA or MVAΔF1L at an M.O.I. of 10 for 8 h or left uninfected and were treated with tetracycline (0.1 µg/ml) for 12 h. Apoptosis was assessed by detection of cytosolic active caspase-3 (data are mean/SEM of n≥3). (PDF) [file ppat.1002083.s003.pdf]

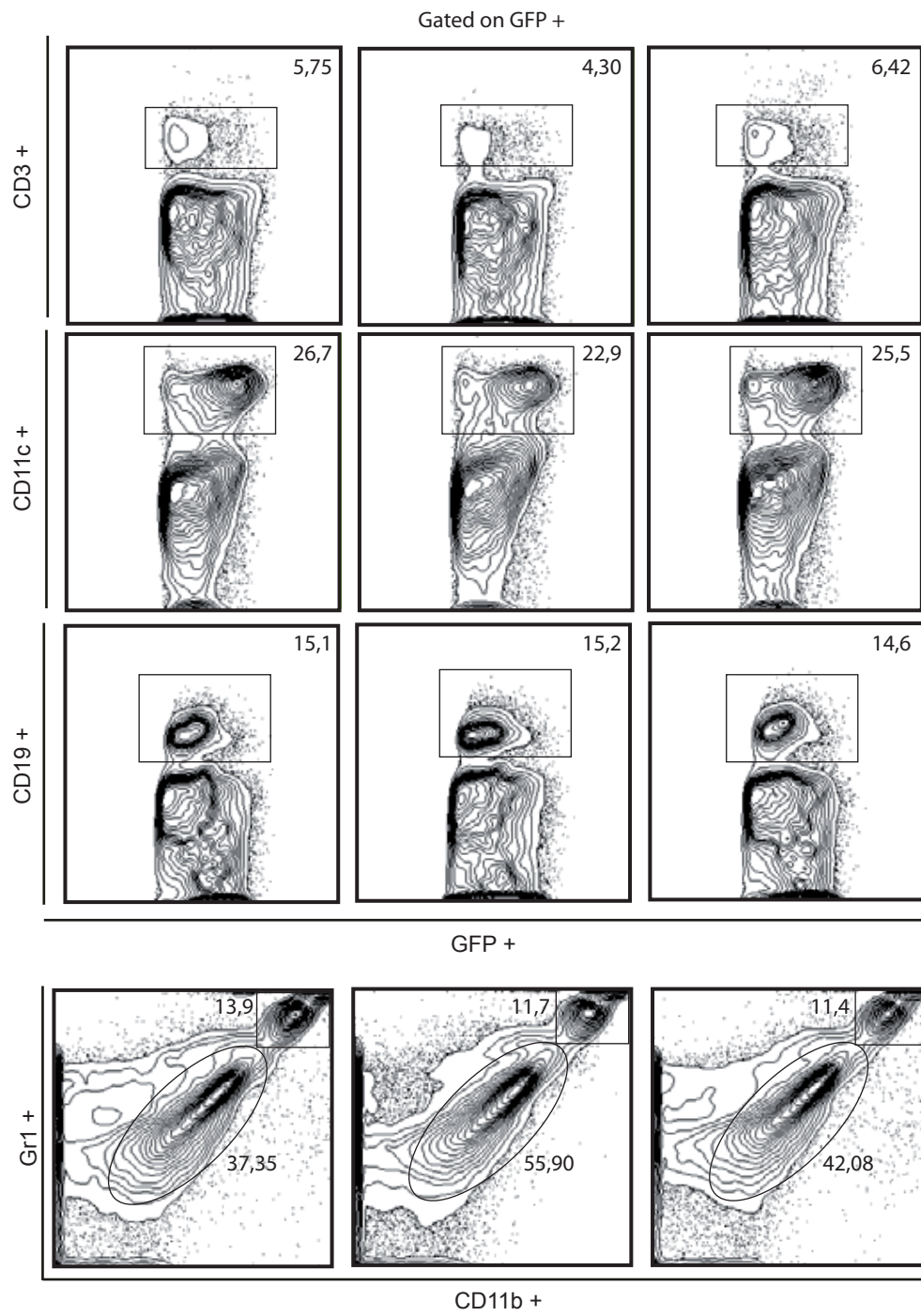

Supplement: Figure S4 — Infection pattern in primary cells upon infection of mice with high-dose MVA. Three mice were in one experiment infected i.v. with 3×10^9^ IU of MVA-GFP 3 h before spleens were harvested. Splenocyte preparations were stained for the markers shown and gated for GFP+ cells for analysis. MVA preferentially infects dendritic cells (CD11c+, second panel from top) and macrophages (CD11b+/Gr1−, bottom). Infection rates of T cells, B cells and granulocytes (CD3, CD19 and CD11b+/Gr-1+) were lower. Each column shows data from one mouse. (PDF) [file ppat.1002083.s004.pdf]

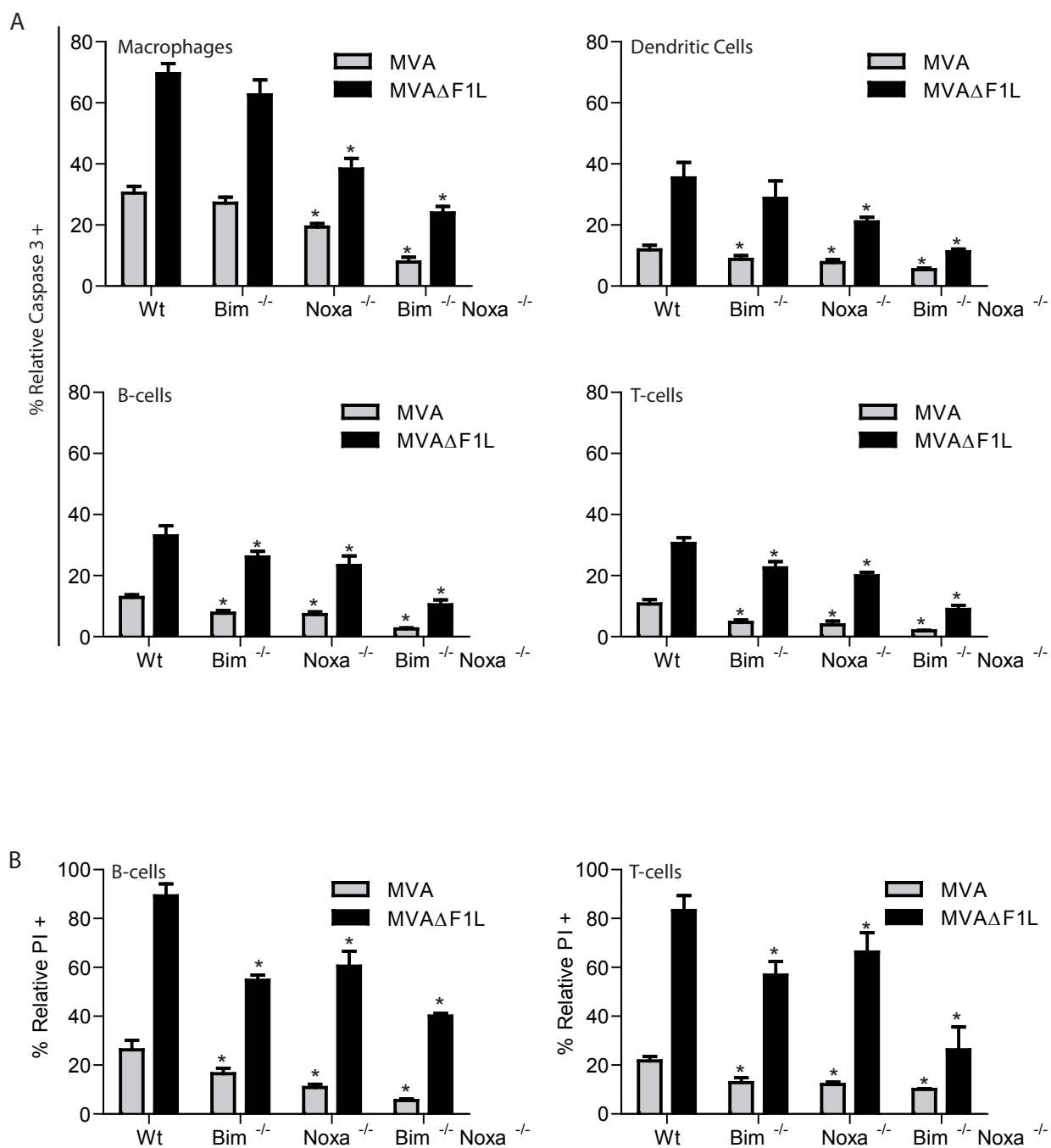

Supplement: Figure S5 — While MVA induced apoptosis is predominantly induced by Noxa, Bim does synergize with Noxa to induce apoptosis in the primary infection target cells of MVA. (A)Samples from Figure 4 were assessed for cytosolic active caspase-3. Active caspase-3 relative numbers were calculated by calculating the % increase in active caspase-3 positive cells proportional to the untreated sample with the following equation: (((% Treated active caspase 3+)−(%untreated active caspase 3+))/(100−% untreated active caspase 3+))*100). (* indicates statistical significance according to the student's t-test, p≤0.05 n≥3). (B) B220+ MACS sorted B-cells and NK1.1−/B220−/MHCII− MACS sorted T-cells were infected with MVA or MVAΔF1L at an M.O.I of 10 for 48 h. Apoptosis was assessed by PI staining and relative numbers were calculated as in Figure 4. (* indicates statistical significance according to the student's t-test, p≤0.05 with data showing mean/SEM of n≥3). (PDF) [file ppat.1002083.s005.pdf]

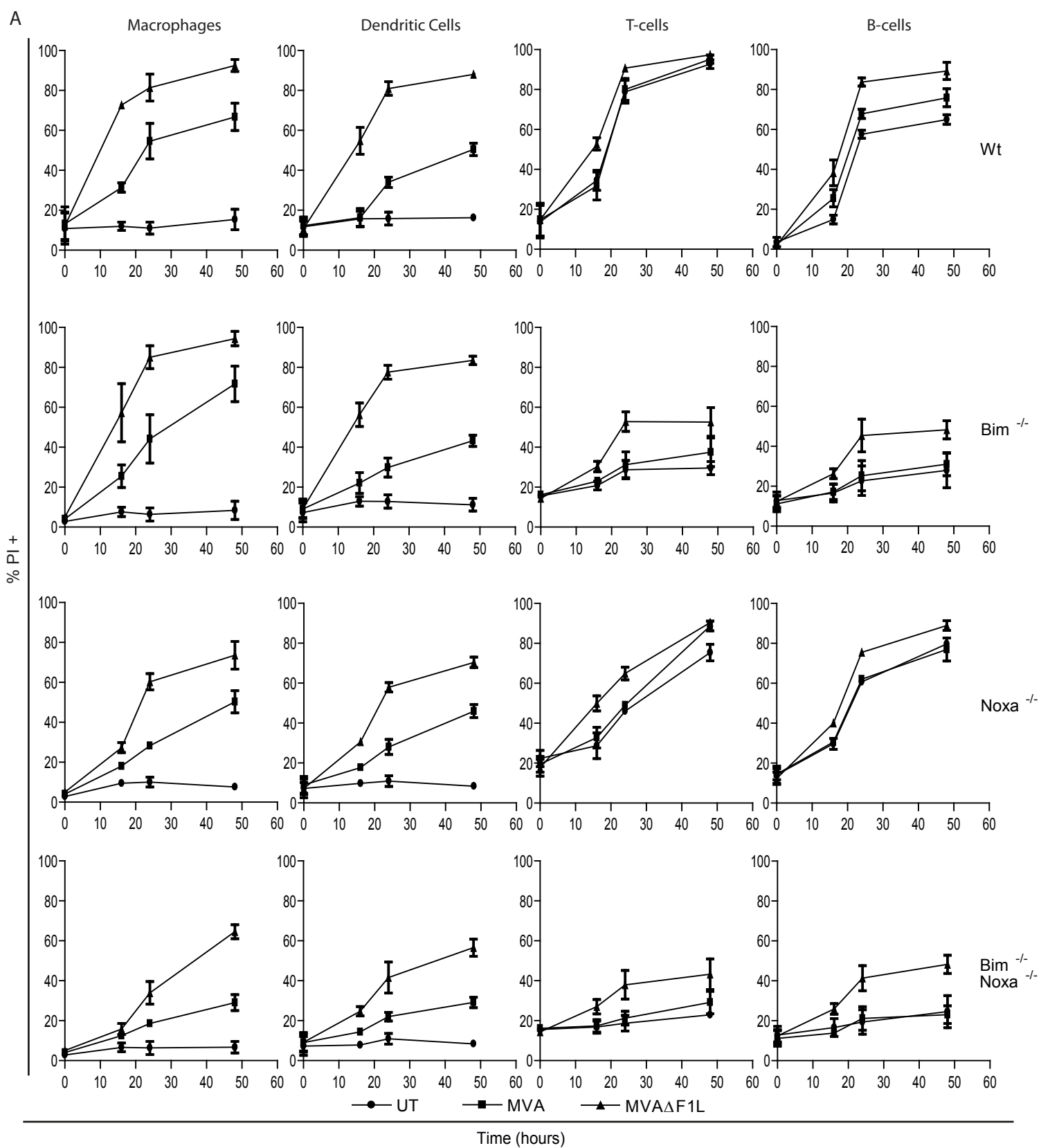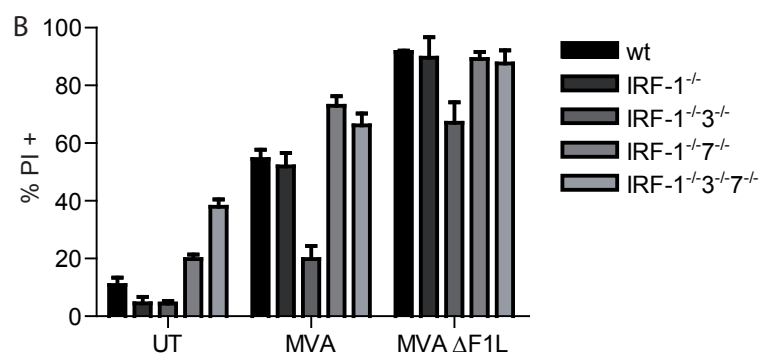

Supplementary Figure S6

Supplement: Figure S6 — Cell death of hematopoietic cells in culture upon viral infection. (A) The same cellular subsets from Figure 4 were assessed by PI staining at the indicated times with or without infection with MVA or MVAΔF1L (n = 3). (B) Macrophages from Figure 5(A) were assessed by PI staining with or without infection with MVA or MVAΔF1L (n = 3). Values represent raw values obtained from three independent experiments without compensating for the simultaneous death of cells in the untreated samples. (PDF) [file ppat.1002083.s006.pdf]

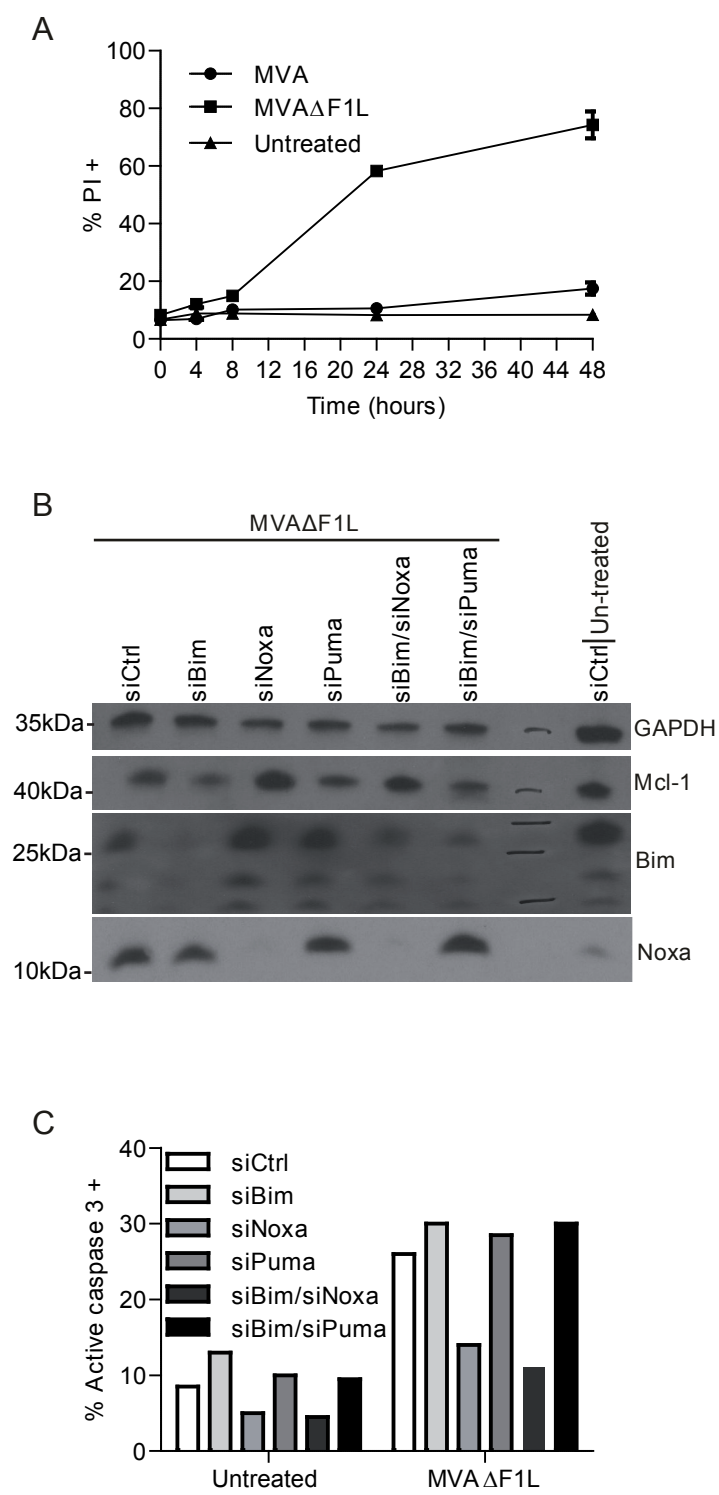

Supplement: Figure S7 — Effect of RNAi to Bim, Noxa and Puma on death during infection of HeLa cells with MVAΔF1L. (A) HeLa cells were infected with MVA or MVAΔF1L at an M.O.I of 10 for the indicated times. Cell death was assessed by PI staining (n≥3). (B) HeLa cells were transfected with the indicated siRNA 24 h prior to infection with MVAΔF1L at an M.O.I of 10 for 20 h. Noxa and Bim levels were assessed before (far right lane) and after infection (all other lanes) (n = 2). (C) Cells from (B) were assessed for apoptosis by detecting cytosolic active caspase-3 (means from n = 2 independent experiments). (* indicates statistical significance according to the student's t-test, p≤0.05 n values indicated). (PDF) [file ppat.1002083.s007.pdf]

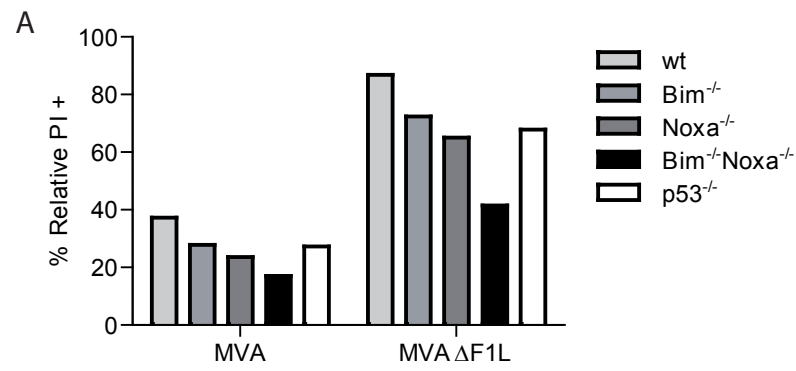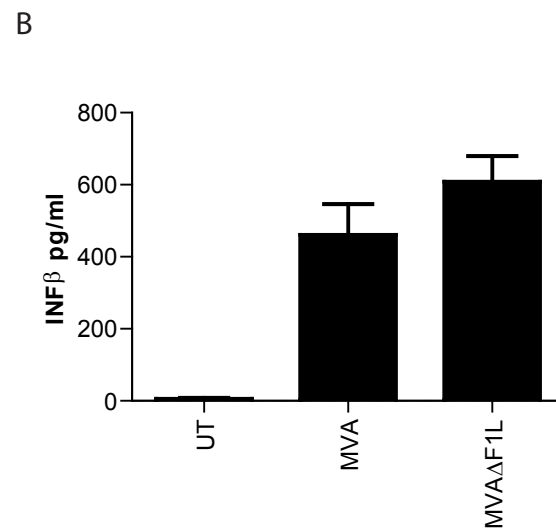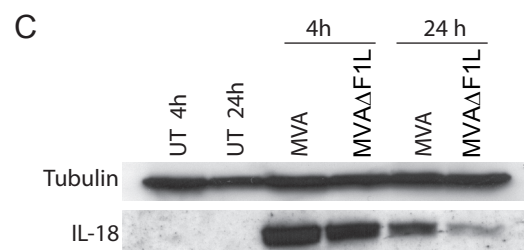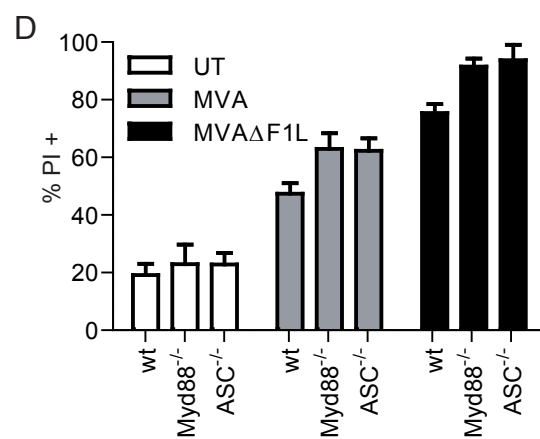

Supplement: Figure S8 — p53, MyD88 and the inflammasome are not required for Noxa mediated apoptosis even though INF-β is produced. (A) M-CSF bone marrow derived macrophages were infected with MVA or MVAΔF1L at an M.O.I of 10 for 20 h. Cell death was assessed by PI staining (means from two independent experiments) (B) IFN-β concentrations were measured by ELISA in supernatants of wt M-CSF bone marrow derived macrophages uninfected or infected with MVA or MVAΔF1L at an M.O.I of 10 for 8 h (n = 3). (C) wt MEFs were infected with MVA or MVAΔF1L at an M.O.I of 10 for the indicated times. Samples were assessed for IL-18 expression by WB (n = 3). (D) M-CSF bone marrow derived macrophages were treated as in A (n = 3). (* indicates statistical significance according to the student's t-test, p≤0.05 n-values indicated). (PDF) [file ppat.1002083.s008.pdf]

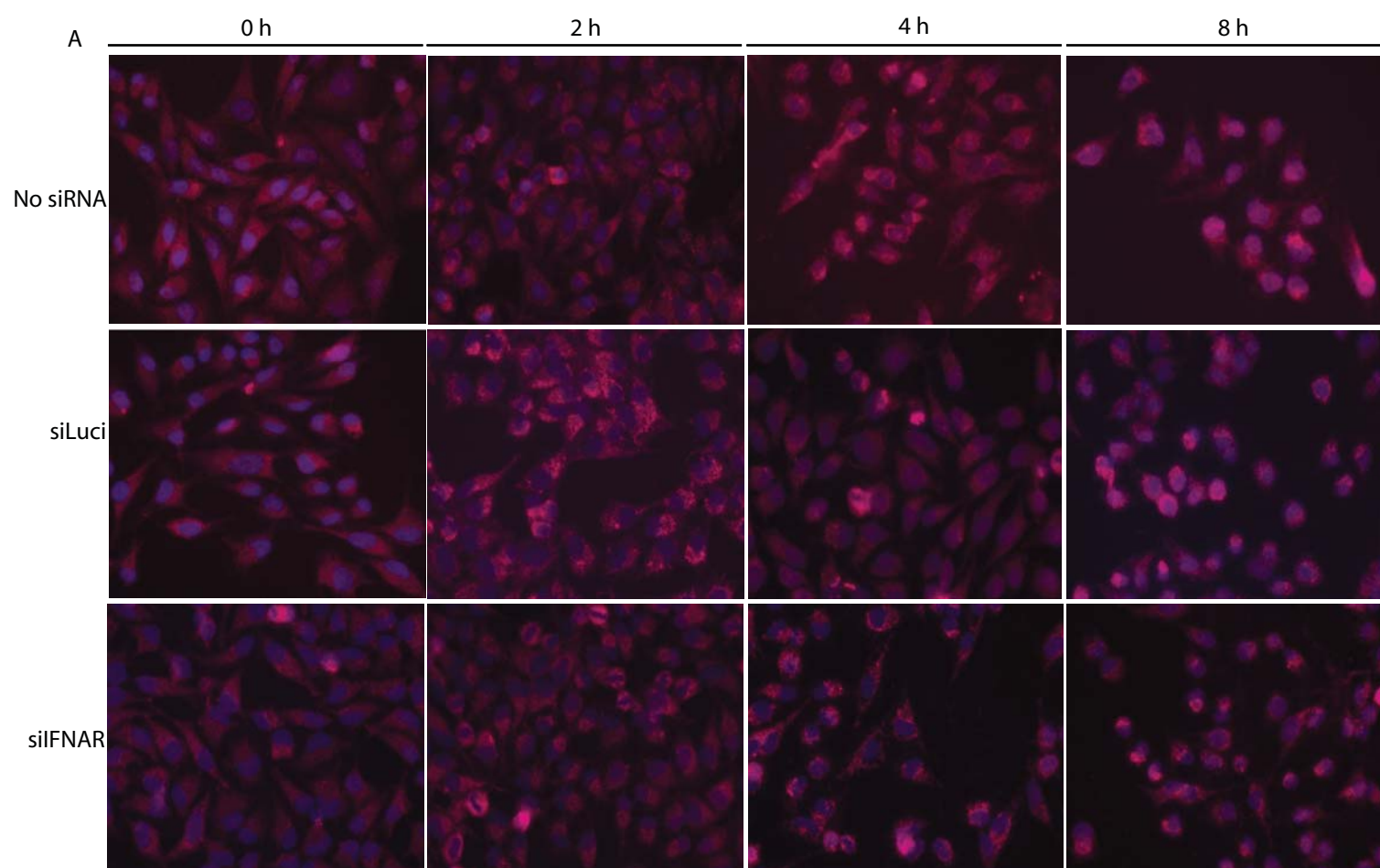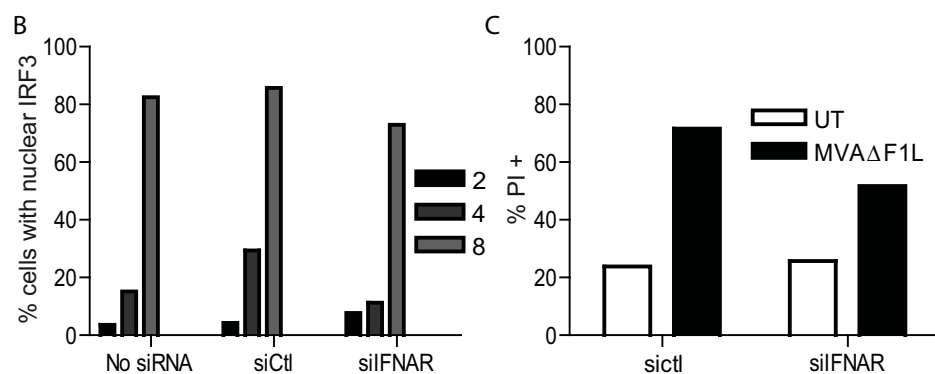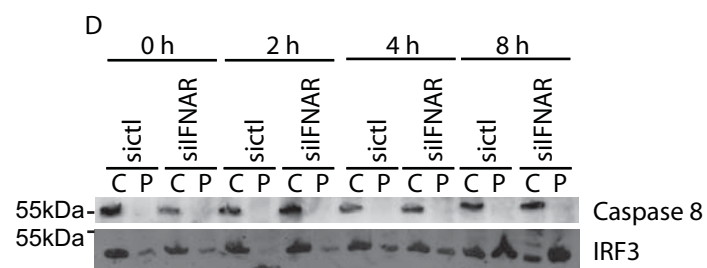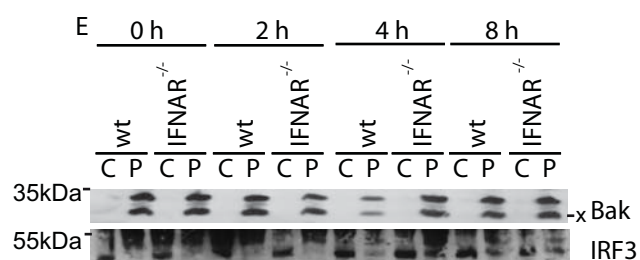

Supplement: Figure S9 — IRF3 translocates to the nucleus in response to MVA infection independently of IFNAR. (A) HeLa cells were transfected with the indicated siRNA 24 h prior to infection with MVA or MVAΔF1L (MOI = 10) for the indicated times. Cells were then fixed and stained with antibody against IRF3 and with Hoechst. The images are representative of the general cellular population at the indicated time points post infection (n = 2). (B) Percentage of cells with nuclear IRF3 were calculated by counting cells in 6 different microscopic fields for each time point and dividing the number of cells with nuclear IRF3 by the total number of cells in the field of view. Data are means of two independent experiments (C) siRNA against IFNAR was functional as it was able to inhibit MVAΔF1L induced apoptosis when cells were infected at an MOI of 10 for 20 h. (D) HeLa cells treated as in (A) were collected at the indicated times, digitonin (0.025% w/v) permeabilized and fractionated into cytosolic and total membrane fractions. IRF3 localization was assessed by Western blot. C is the cytosolic fraction released upon cellular membrane permeabilization. P represents the pellet fraction including nucleus, cellular membrane and intact mitochondria. (E) wt and IFNAR−/− ER-HoxB8 immortalized macrophages were infected with MVA or MVAΔF1L at an MOI of 10 for the indicated time points after 7 days differentiation in GM-CSF medium deficient in β-estridiol, and IRF3-translocation was determined as in (D). (PDF) [file ppat.1002083.s009.pdf]

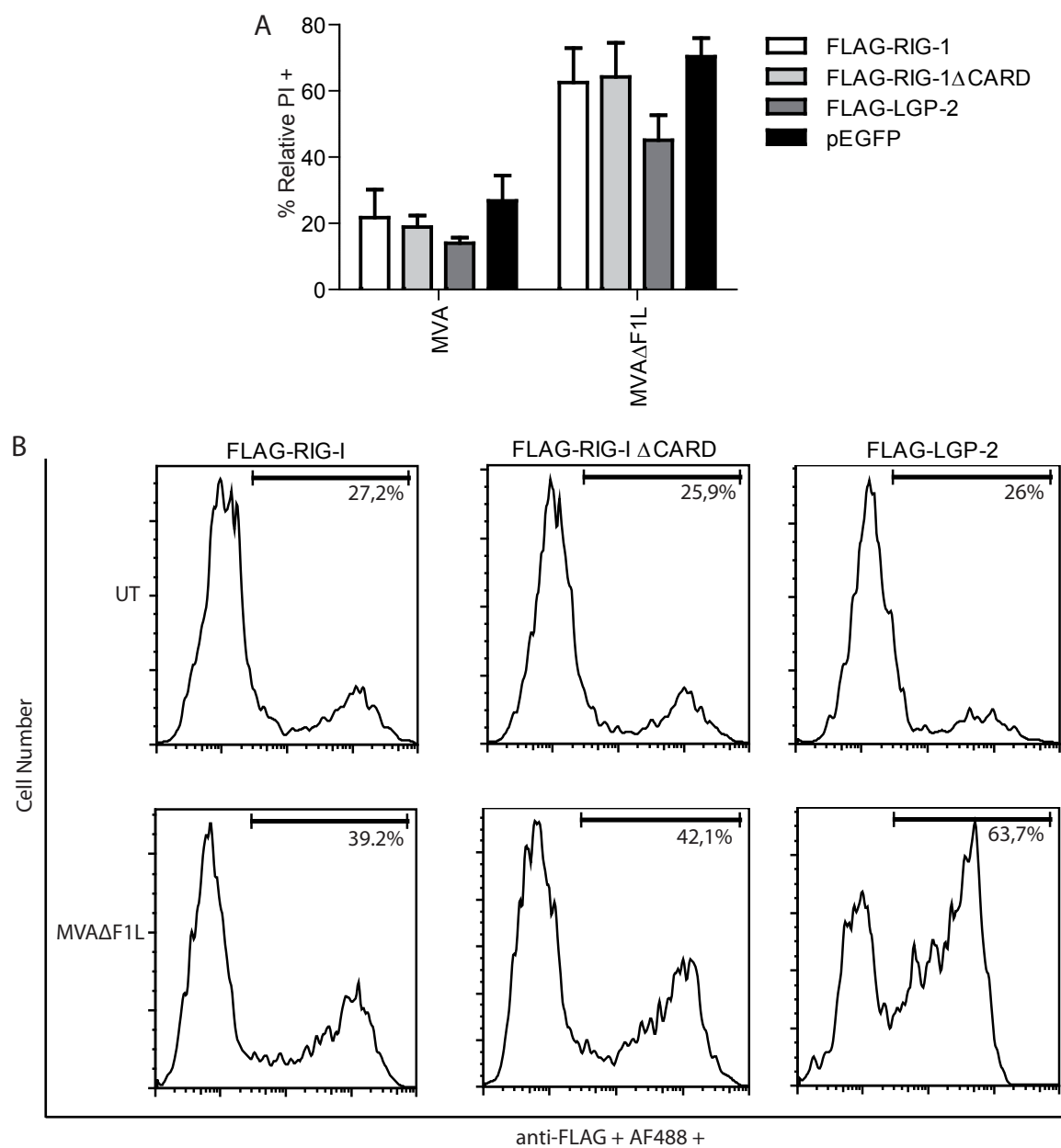

Supplement: Figure S10 — LGP2 can act as a dominant negative inhibitor of MVAΔF1L induced apoptosis. (A) pEF-BOS plasmids containing the indicated FLAG-tagged proteins or pEGFP (transfection control) were transfected into HeLa cells. After 24 h cells were infected with MVA or MVAΔF1L at an M.O.I of 10 for 20 h. Cell death was assessed by PI staining and relative numbers were calculated as in Figure 4 (n = 3). (B) Samples from A were stained for intracellular FLAG before and after infection with MVAΔF1L at an M.O.I of 10 for 20 h (n = 2). (PDF) [file ppat.1002083.s010.pdf]

A

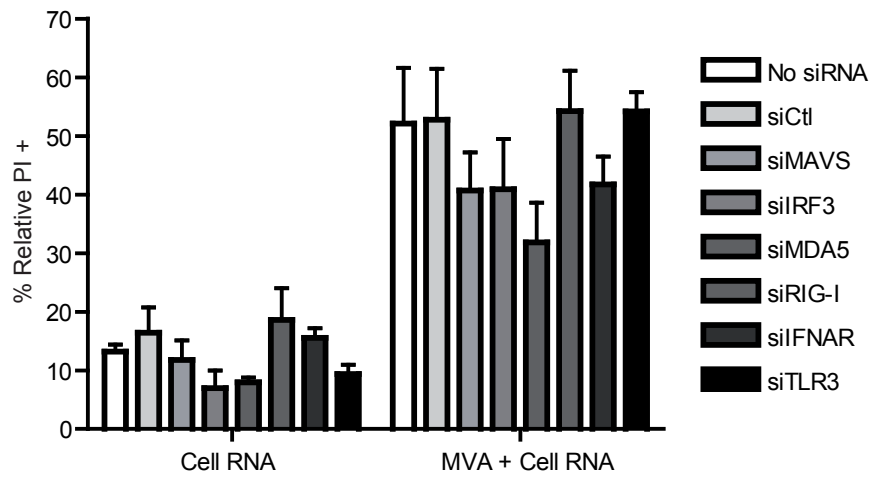

B

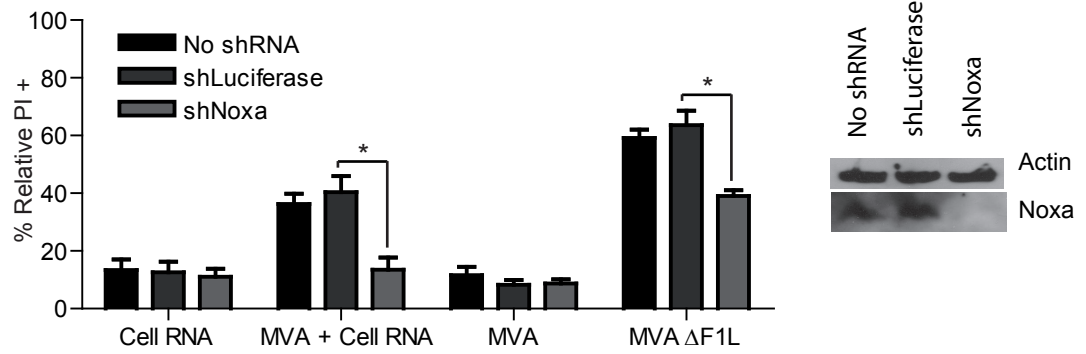

Supplement: Figure S11 — Full viral RNA induced apoptosis requires RNA helicase dependent induction of Noxa. (A) HeLa cells were transfected with the indicated siRNA 24 h prior to transfection of total RNA from HeLa cells infected with MVA or uninfected HeLa cells using Fugene reagent. Cell death was assessed 24 h later by PI staining and relative numbers were calculated by calculating the % increase in PI proportional to the Fugene-alone treated sample as in Figure 7 (with data showing mean/SEM of n≥3). (B) Hela cells stably expressing shRNA specific to Luciferase (control) and Noxa were transfected with total RNA from HeLa cells infected with MVA or uninfected HeLa cells using Fugene reagent; or infected with MVA or MVAΔF1L. Cell death was assessed 24 h later by PI staining and relative numbers were calculated by calculating the % increase in PI proportional to the Fugene-alone treated sample as in Figure 7. In the case of infection with MVA or MVAΔF1L relative values were calculated as in Figure 4 (with data showing mean/SEM of n = 3). shRNA efficiency was assessed by Western Blot (right hand panel) (* indicates statistical significance according to the student's t-test, p≤0.05 with data indicating mean/SEM of n≥3; x indicates unspecific band). (PDF) [file ppat.1002083.s011.pdf]
